# Prenatal Remote Monitoring of Women With Gestational Hypertensive Diseases: Cost Analysis

**DOI:** 10.2196/jmir.9552

**Published:** 2018-03-26

**Authors:** Dorien Lanssens, Thijs Vandenberk, Christophe JP Smeets, Hélène De Cannière, Sharona Vonck, Jade Claessens, Yenthel Heyrman, Dominique Vandijck, Valerie Storms, Inge M Thijs, Lars Grieten, Wilfried Gyselaers

**Affiliations:** ^1^ Mobile Health Unit Faculty of Medicine and Life Sciences Hasselt University Hasselt Belgium; ^2^ Department of Gynaecology Ziekenhuis Oost-Limburg Genk Belgium; ^3^ Department of Cardiology Ziekenhuis Oost-Limburg Genk Belgium; ^4^ Department of Health and Life Sciences Hasselt University Hasselt Belgium; ^5^ Faculty of Medicine and Life Sciences Ghent University Gent Belgium; ^6^ Future Health Department Ziekenhuis Oost-Limburg Genk Belgium; ^7^ Department of Physiology Hasselt University Hasselt Belgium

**Keywords:** remote monitoring, gestational hypertensive diseases, reimbursement, cost-effectiveness

## Abstract

**Background:**

Remote monitoring in obstetrics is relatively new; some studies have shown its effectiveness for both mother and child. However, few studies have evaluated the economic impact compared to conventional care, and no cost analysis of a remote monitoring prenatal follow-up program for women diagnosed with gestational hypertensive diseases (GHD) has been published.

**Objective:**

The aim of this study was to assess the costs of remote monitoring versus conventional care relative to reported benefits.

**Methods:**

Patient data from the Pregnancy Remote Monitoring (PREMOM) study were used. Health care costs were calculated from patient-specific hospital bills of Ziekenhuis Oost-Limburg (Genk, Belgium) in 2015. Cost comparison was made from three perspectives: the Belgian national health care system (HCS), the National Institution for Insurance of Disease and Disability (RIZIV), and costs for individual patients. The calculations were made for four major domains: prenatal follow-up, prenatal admission to the hospital, maternal and neonatal care at and after delivery, and total amount of costs. A simulation exercise was made in which it was calculated how much could be demanded of RIZIV for funding the remote monitoring service.

**Results:**

A total of 140 pregnancies were included, of which 43 received remote monitoring (30.7%) and 97 received conventional care (69.2%). From the three perspectives, there were no differences in costs for prenatal follow-up. Compared to conventional care, remote monitoring patients had 34.51% less HCS and 41.72% less RIZIV costs for laboratory test results (HCS: mean €0.00 [SD €55.34] vs mean €38.28 [SD € 44.08], *P*<.001; RIZIV: mean €21.09 [SD €27.94] vs mean €36.19 [SD €41.36], *P*<.001) and a reduction of 47.16% in HCS and 48.19% in RIZIV costs for neonatal care (HCS: mean €989.66 [SD €3020.22] vs mean €1872.92 [SD €5058.31], *P*<.001; RIZIV: mean €872.97 [SD €2761.64] vs mean €1684.86 [SD €4702.20], *P*<.001). HCS costs for medication were 1.92% lower in remote monitoring than conventional care (mean €209.22 [SD €213.32] vs mean €231.32 [SD 67.09], *P*=.02), but were 0.69% higher for RIZIV (mean €122.60 [SD €92.02] vs mean €121.78 [SD €20.77], *P*<.001). Overall HCS costs for remote monitoring were mean €4233.31 (SD €3463.31) per person and mean €4973.69 (SD €5219.00) per person for conventional care (*P*=.82), a reduction of €740.38 (14.89%) per person, with savings mainly for RIZIV of €848.97 per person (23.18%; mean €2797.42 [SD €2905.18] vs mean €3646.39 [SD €4878.47], *P*=.19). When an additional fee of €525.07 per month per pregnant woman for funding remote monitoring costs is demanded, remote monitoring is acceptable in their costs for HCS, RIZIV, and individual patients.

**Conclusions:**

In the current organization of Belgian health care, a remote monitoring prenatal follow-up of women with GHD is cost saving for the global health care system, mainly via savings for the insurance institution RIZIV.

## Introduction

Remote monitoring in obstetrics is a relatively new field of research; only a few trials have shown the effectiveness of remote monitoring in obstetrical care for both mother and child. When uterine activity is transmitted by telecommunication, significant prolonged pregnancy survivals are observed [1,2]. Higher feelings of self-efficacy and a reduction in (unscheduled) face-to-face visits [3-6] is reported when remote monitoring is used in the prenatal follow-up of pregnant women with gestational diabetes mellitus in comparison to conventional care. In addition, elevated feelings of maternal satisfaction were obtained when remote monitoring was used in obstetrical care [3,6-8]. Finally, the newborns did have a higher gestational age at delivery [9] and were less likely to be of low birth weight [1,9] or to be admitted to the neonatal intensive care unit [1,9,10] when a remote monitoring group was compared to a conventional care group. In an earlier publication, we reported that remote monitoring in pregnant women with gestational hypertensive diseases (GHD) reduces the number of inductions and maternal prenatal admissions [10]. However, until now, few studies have evaluated the economic impact of remote monitoring compared to conventional care [9,11], and no study is known about the cost-effectiveness of a remote monitoring prenatal follow-up program for women diagnosed with GHD.

The Pregnancy Remote Monitoring (PREMOM) study was designed for women diagnosed with GHD who had their prenatal follow-up in Ziekenhuis Oost-Limburg (Genk, Belgium). According to the Flanders’ register of perinatal outcomes, the prevalence of hypertensive disorders in pregnancy is 4.6%: 0.3% deliver before 34 weeks, 0.6% deliver between 34 and 37 weeks, and 3.7% deliver after 37 weeks [12]. As a continuation of this trial, a study was designed with the objective of quantifying the costs of both remote monitoring and conventional care from the perspectives of the Belgium global health care system (HCS), which combines costs for the National Institution for Insurance of Disease and Disability (*Rijksinstituut voor Ziekte- en Invaliditeitsverzekering*; RIZIV) and costs for individual patients [13]. The calculations were made for four major domains: prenatal follow-up, prenatal admission to the hospital, maternal and neonatal care at and after delivery, and total amount of costs. A simulation exercise was made when an additional fee of €100 per month per patient for remote monitoring was charged. We hypothesized the addition of remote monitoring to a prenatal follow-up program for pregnant women with GHD to be cost-effective when compared to conventional care. This paper reports on the results for the Belgium situation.

## Methods

### Data

Data collected from the PREMOM study was used for this cost analysis. The PREMOM study design and data collection method are described in detail elsewhere [10]. Briefly, the PREMOM study was a 1-year retrospective study, performed in the outpatient clinic of a second-level prenatal center where pregnant women with GHD received remote monitoring or conventional care. From January 1 to December 31, 2015, 166 pregnant women were diagnosed with GHD: 53 of them received remote monitoring and 113 received conventional care. After excluding five patients in the remote monitoring group and 15 in the conventional care group because of missing data, 48 patients in the remote monitoring group and 98 in the conventional care group were included in the final analysis.

Women consenting for remote monitoring received obstetric surveillance using a Withings Wireless Blood Pressure Monitor, Withings Smart Body Analyzer, and a Withings Pulse O_2_ (Withings, Issy-les-Moulineux, France). Pregnant women participating in the prenatal remote follow-up program were asked to perform one blood pressure measurement in the morning and one in the evening, one weight measurement a day, and to wear an activity tracker day and night until delivery or hospital admission. The data from the monitor devices were transmitted to a Web-based dashboard developed by the mobile health unit of Hasselt University. Predetermined alarm signals were set and alarm events were communicated with the obstetrician in charge to discuss management options before contacting and instructing patients at home. Therapeutic interventions were according to local management. The clinical goal of routine prenatal outpatient care is to timely detect an abnormal course of maternal and/or fetal health. The study protocol was approved by the local ethics committees responsible for the site. The investigation conformed to the principles outlined in the Declaration of Helsinki. All patients gave written informed consent, and data were treated confidentially.

### Study Design

The objective of the study was to quantify the costs of remote monitoring versus conventional care from the perspectives of the HCS, the RIZIV, and the patients. The costs of the HCS are the total amount of costs that have to be paid to cover the care that has been provided. These HCS costs can be divided into two subgroups who have to pay their part of the costs: (1) RIZIV, the national institutional social security in Belgium, which ensures every insured individual, regardless of financial situation, has access to necessary qualitative medical care in accordance with the tariff agreements between caregivers and government [14] and (2) the patients who have to pay their part of care from their own financial resources. The HCS costs are estimated by using the national tariffs applied for these services. The costs for the RIZIV were calculated using the Belgium national reimbursement tariffs [12]. The costs for the patients were the HCS costs minus the RIZIV costs. The four major domains in which the costs are divided and their subcategories are presented subsequently. A detailed overview of the included costs are presented in Multimedia Appendix 1.

#### Cost Analysis

##### Prenatal Follow-Up

All costs related to urgent and nonurgent in-office visits were used in the prenatal follow-up cost analysis: (1) costs of prenatal consultations, (2) costs of ultrasounds, and (3) costs of cardiotocographics.

##### Prenatal Admission to the Hospital

To evaluate the economic impact of remote monitoring on the three major stakeholders, the following data points were collected when the pregnant women were admitted to the prenatal ward: (1) costs related to the laboratory test results of the mother, (2) costs of the medicines, and (3) costs related to the admission.

##### Maternal and Neonatal Care at and After Delivery

For both groups, the following costs were included for this topic: (1) costs of the delivery, (2) costs necessary for the care of the neonate, and (3) other costs.

##### Total Amount of Costs

After analyzing the previously mentioned data, a cost analysis of the total amount of costs was made. This included (1) costs of the prenatal follow-up, (2) costs of the prenatal admission to the prenatal ward, and (3) costs of the maternal and neonatal care at and after delivery.

### Simulation Exercise

A simulation exercise was made in which the amount that could be demanded by RIZIV for funding of the remote monitoring service was calculated. This charge was calculated by dividing the cost savings in RIZIV (by subtracting the total costs of the remote monitoring group from those of the conventional care group) by the mean time of prenatal remote monitoring follow-up per pregnant woman. This charge could be used to finance the costs which were needed to perform remote monitoring in the prenatal follow-up of women at risk for GHD, such as the need of midwives to accompany the pregnant women to their remote monitoring follow-up and to interpret the (alarm) signals, the need of obstetrics to refer and supervise the pregnant women at risk, and the need of technical staff to maintain the platform, to give technical support, etc.

### Statistical Analysis

The baseline characteristics are continuous data summarized as mean and standard deviation. Categorical data are summarized as count and percentage and were compared using the chi-square test or Fisher exact test, when appropriate. Costs were reported as means, standard deviations, medians, and interquartile ranges. Cost data are typically highly skewed [14] because a few patients incur particularly high costs; therefore, the Mann-Whitney *U* test was used to compare costs across groups. Both univariate and multivariate analyses were performed for analyzing the costs for the three domains.

The nominal level alpha<.05 was considered significant. All statistical analyses were performed with SPSS release 24.0.

## Results

### Baseline Characteristics

The baseline characteristics of the patients are summarized in Table 1. Of the 48 patients participating in the remote monitoring study, five (10%) were excluded due to missing data. In the conventional care group, one participant was excluded due to missing data (1/98, 1%). Finally, the remote monitoring group consisted of 43 (30.7%) patients and the conventional care group had 97 (69.3%). The baseline clinical characteristics of the population enrolled were almost homogeneous, without differences between the two groups except for primigravida (44%, 19/43) in the remote monitoring group versus 66% (65/97) in the conventional care group (*P*=.02) and smoking (0%, 0/43) in the remote monitoring group versus 10%, (10/97) in the conventional care group (*P*=.03).

**Table 1:** Baseline clinical characteristics (N=140).

Variables	Remote monitoring group (n=43)	Conventional care group (n=97)	*P* value (2-tailed)
Age (years), mean (SD)	31.72 (4.44)	31.95 (4.77)	.77
Prepregnancy weight (kg), mean (SD)	70.12 (16.26)	76.80 (19.75)	.05
Height (cm), mean (SD)	165.65 (6.89)	167.08 (6.86)	.18
BMI (kg/m^2^), mean (SD)	25.23 (5.03)	27.01 (6.94)	.32
Primigravida, n (%)	19 (44)	65 (66)	.02
Cardiovascular disorders, n (%)	0 (0)	1 (1)	.99
Coagulation disorders, n (%)	1 (2)	1 (1)	.52
Endocrine disorders, n (%)	2 (5)	5 (5)	.99
Immunology disorders, n (%)	1 (2)	2 (2.04)	.99
Smoker, n (%)	0 (0)	10 (10)	.03

### Health Care Costs

The health care costs are presented in Table 2. The results are discussed in detail subsequently.

To investigate the influence of the maternal demographics and characteristics on the health care costs, a multiple linear regression analysis and a multivariate logistic regression analysis was performed. A detailed overview of these data are provided in Multimedia Appendix 2. No important influences of the maternal demographics and characteristics was found in the health care costs.

### Cost Analysis

#### Prenatal Follow-Up

No differences were found in costs for prenatal follow-up (prenatal visits, ultrasounds, and costs of cardiotocographics): not in the costs for the HCS, the RIZIV, or the patients.

#### Prenatal Admission to the Hospital

Patients admitted to the remote monitoring group did have 34.51% less HCS and 41.72% less RIZIV costs for laboratory test results compared to conventional care group (HCS: remote monitoring mean €25.07, SD €55.34 vs conventional care mean €38.28, SD €44.08, *P*<.001; RIZIV: remote monitoring mean €21.09, SD €27.94 vs conventional care mean €36.19, SD €41.36, *P*<.001). Also, the HCS cost for the medicaments were 1.92% lower in the remote monitoring group compared to the conventional care group (mean €209.22, SD €141.86 vs mean €213.32, SD €67.09, *P*=.02), but the RIZIV costs were 0.69% higher in the remote monitoring group compared to the conventional care group (mean €122.60, SD €92.02 vs mean €121.76, SD €20.77, *P*<.001).

#### Maternal and Neonatal Care at and After Delivery

No differences were found in costs for delivery in the remote monitoring group versus the conventional care group. A reduction of 47.16% in HCS cost and 48.19% in RIZIV costs for neonatal care was found in the remote monitoring group compared to the conventional care group (HCS: remote monitoring mean €989.66, SD €3020.22 vs conventional care mean €1872.92, SD €5058.31, *P*<.001; RIZIV: remote monitoring mean €872.97, SD €2761.64 vs conventional care mean €1684.86, SD €4702.20, *P*<.001). Other costs were for the HCS 57.86% and RIZIV 58.63% lower in remote monitoring versus conventional care (HCS: remote monitoring mean €26.63, SD €11.83 vs conventional care mean €63.19, SD €158.23, *P*=.04; RIZIV remote monitoring mean €26.14, SD €19.86 vs conventional care mean €63.19, SD €158.23, *P*<.001), but 0.77% higher for the patients in remote monitoring versus conventional care (mean €0.49, SD €20.99 vs mean €0.00, SD €0.00, *P*=.01).

### Total Amount of Costs

**Figure 1:**
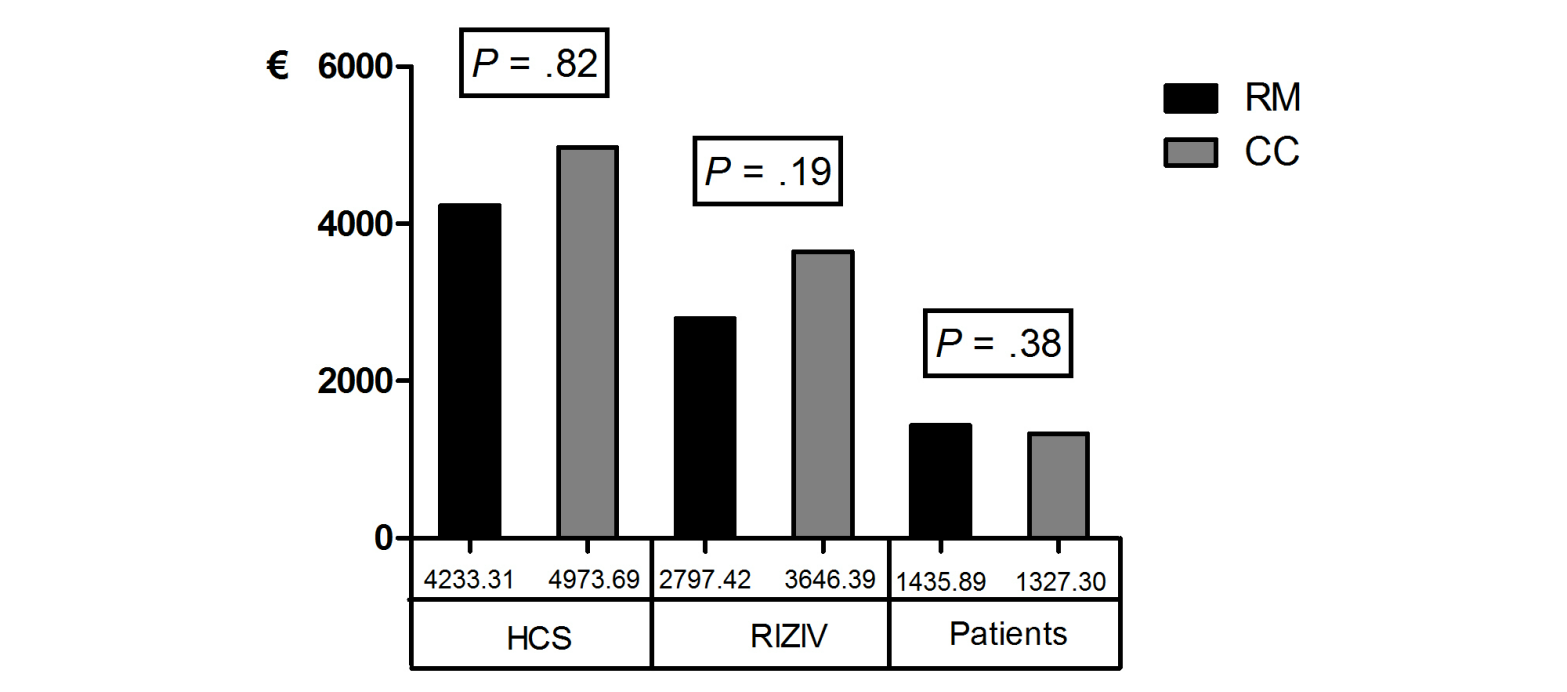
Total amount of costs for remote monitoring (RM) and conventional care (CC) groups paid by health care service (HCS), National Institution for Insurance of Disease and Disability (RIZIV), and patients.

An overview of the total amount of costs is presented in Figure 1 and in Multimedia Appendix 3. There were no significant differences between remote monitoring and conventional care in total amount of costs for HCS (remote monitoring mean €4233.31, SD €3463.31 vs conventional care mean €4973.69, SD €5219.00, *P*=.82), the RIZIV (remote monitoring mean €2797.42, SD €2905.18 vs conventional care mean €3646.40, SD 4878.47, *P*=.19), or the patients (remote monitoring mean €1435.89, SD €829.09 vs conventional care mean €1327.30, SD €753.94, *P*=.38). But, a cost reduction of €740.38 per person (14.89%) was made for HCS and a cost reduction of €848.97 (23.18%) was made for RIZIV in remote monitoring compared to conventional care. Patient’s costs were slightly higher (€108.59, 8.18%) for remote monitoring than for conventional care.

### Simulation Exercise

**Figure 2:**
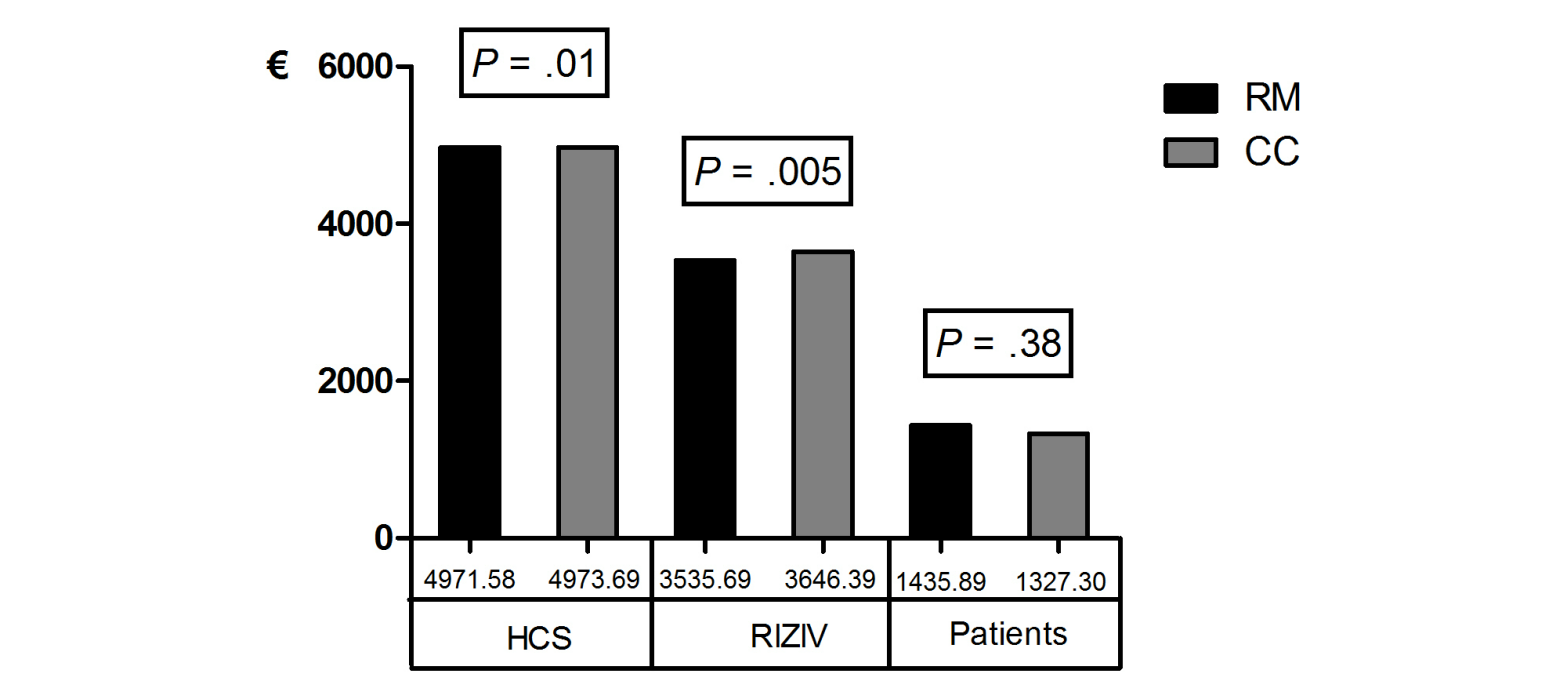
Total amount of costs plus remote monitoring for remote monitoring (RM) and conventional care (CC) groups paid by health care service (HCS), National Institution for Insurance of Disease and Disability (RIZIV), and patients.

A simulation exercise was made in which it was calculated how much could be demanded of RIZIV for funding the remote monitoring service. For this study, 43 pregnant women were included in the analysis with a range of 1 day of participation to 145 days of participation in the PREMOM project. The mean time of participation in this project was 44.42 days or 1.41 months (Multimedia Appendix 4). By dividing €740.35 by 1.41 months, a funding of €525.07 per month per pregnant woman could be asked. Because of the difference of almost €1000 per person in costs for the RIZIV, it was reasonable to charge the supplementary costs to RIZIV. As a result, there was a significant difference in costs for HCS of a reduction of €2.11 per person in remote monitoring versus conventional care (remote monitoring: mean €4971.58, SD 3479.69; conventional care: mean 4973.69, SD 5219.00, *P*=.01) and in RIZIV costs by also having a reduction of €110.70 per person in remote monitoring versus conventional care (remote monitoring: mean 3535.69, SD 2931.90; conventional care: mean 3646.39, SD 4878.47, *P*=.005). The patient still does not have to pay more for their prenatal care (remote monitoring: mean €1435.89, SD €829.09; conventional care: mean €1327.30, SD €753.94, *P*=.38). An overview of the costs is shown in Multimedia Appendix 4 and in Figure 2.

**Table 2:** Health care costs. All costs in euros. HCS: health care system; IQR: interquartile range; RIZIV: National Institution for Insurance of Disease and Disability.

Cost variable			Study group					Cost savings in Euros in remote monitoring group, n (%)		*P* value (2-tailed)
			Remote monitoring (n=43)		Conventional care (n=97)							
			Mean (SD)	Median (IQR)	Mean (SD)	Median (IQR)					
**Prenatal follow-up**										
	**Prenatal visits**									
		HCS	184.26 (79.10)	205.80 (144.06-226.38)	183.31 (71.79)	185.22 (144.06-226.38)	–0.95 (–0.52)		.71	
		RIZIV	110.58 (47.83)	123.50 (86.45-135.85)	110.00 (43.08)	111.15 (86.45-135.85)	–0.58 (–0.52)		.71	
		Patients	73.69 (31.87)	82.30 (57.61-90.53)	73.31 (28.71)	74.07 (57.61-90.53)	–0.38 (–0.52)		.71	
	**Ultrasounds**									
		HCS	89.66 (58.61)	79.77 (79.77-106.36)	96.49 (57.23)	79.77 (79.77-106.36)	6.83 (7.08)		.96	
		RIZIV	81.30 (53.14)	72.33 (72.33-96.44)	87.49 (51.89)	72.33 (72.33-96.44)	6.19 (7.08)		.96	
		Patients	8.36 (5.47)	7.44 (7.44-9.92)	9.00 (5.34)	7.44 (7.44-9.92)	0.64 (7.08)		.96	
	**Cardiotocographics**									
		HCS	127.58 (130.45)	124.68 (0.00-187.02)	93.19 (105.37)	62.34 (0.00-124.68)	–34.39 (–36.90)		.15	
		RIZIV	63.79 (65.22)	62.34 (0.00-93.1)	46.59 (52.68)	31.17 (0.00-62.34)	–17.20 (–36.90)		.15	
		Patients	63.79 (65.22)	62.34 (0.00-93.51)	46.59 (52.68)	31.17 (31.17-62.34)	–17.20 (-36.90)		.15	
**Prenatal admission**										
	**Laboratory test results**									
		HCS	25.07 (55.34)	0.00 (0.00-19.58)	38.28 (44.08)	27.86 (5.13-56.74)	13.21 (34.51)		<.001	
		RIZIV	21.09 (27.94)	0.00 (0.00-19.07)	36.19 (41.36)	25.74 (5.13-50.53)	15.10 (41.72)		<.001	
		Patients	3.98 (14.06)	0.00 (0.00-0.00)	2.09 (8.78)	0.00 (0.00-0.00)	–1.89 (–90.43)		.78	
	**Prenatal admission**									
		HCS	1423.57 (1184.78)	1166.62 (1013.25-1407.54)	1336.40 (670.99)	1172.61 (950.68-1450.04)	–87.17 (–6.52)		.73	
		RIZIV	798.47 (596.93)	663.30 (600.25-786.59)	783.44 (372.81)	714.96 (501.09-922.33)	–15.03 (–1.92)		.63	
		Patients	625.10 (606.57)	497.67 (394.29-617.61)	552.96 (372.50)	477.88 (324.57-663.41)	–72.14 (–13.05)		.41	
	**Medicaments**									
		HCS	209.22 (141.86)	168.73 (155.71-206.18)	213.32 (67.09)	204.65 (168.99-233.79)	4.10 (1.92)		.02	
		RIZIV	122.60 (92.02)	106.03 (99.61-111.77)	121.76 (20.77)	114.81 (108.02-130.01)	–0.84 (–0.69)		<.001	
		Patients	86.61 (68.81)	63.71 (47.69-97.87)	91.56 (20.77)	79.13 (55.67-108.43)	4.95 (5.41)		.14	
**Maternal and neonatal care**										
	**Delivery**									
		HCS	1157.66 (469.34)	1298.10 (670.34-1329.38)	1076.61 (485.14)	998.94 (670.34-1298.10)	–81.05 (–7.53)		.15	
		RIZIV	700.48 (186.41)	670.34 (370.34-685.98)	712.87 (196.03)	670.34 (663.34-755.66)	12.39 (1.74)		.79	
		Patients	457.17 (344.53)	627.76 (0.00-643.40)	363.73 (404.17)	424.11 (0.00-628.86)	–93.44 (–25.69)		.15	
	**Neonatal care**									
		HCS	989.66 (3020.22)	146.32 (102.67-374.19)	1872.92 (5058.31)	290.78 (147.69-625.23)	883.26 (47.16)		<.001	
		RIZIV	872.97 (2761.64)	98.48 (85.49-279.14)	1684.86 (4702.20)	230.45 (104.81-519.38)	811.89 (48.19)		<.001	
		Patients	116.69 (263.74)	48.22 (13.01-95.05)	188.06 (413.95)	61.68 (23.69-120.19)	71.37 (37.95)		.10	
	**Other**									
		HCS	26.63 (11.83)	25.73 (25.73-25.73)	63.19 (158.23)	25.73 (25.73-25.73)	36.56 (57.86)		.04	
		RIZIV	26.14 (19.86)	25.73 (21.10-25.73)	63.19 (158.23)	25.73 (25.73-25.73)	37.05 (58.63)		<.001	
		Patients	0.49 (20.99)	0.00 (0.00-0.00)	0.00 (0.00)	25.73 (25.73-25.73)	–0.49 (–0.77)		.01	

## Discussion

### Principal Findings

The main finding of this study is that a remote monitoring prenatal follow-up for pregnant women at risk for GHD reduces the total amount of costs for national health care in comparison to a standard follow-up strategy. This cost reduction is due to a marked reduction in the consumption of health care services, including laboratory test results taken, medication use, and maternal and neonatal admissions. When an additional fee of €525.07 per month per pregnant woman for funding remote monitoring costs is asked, remote monitoring is still acceptable in their costs for HCS, RIZIV, and individual patients.

### Strengths and Limitations

The use of “real-life” data from the hospital bills is the main strength of this study. By using these data, the actual situation of pregnancies complicated with GHD is simulated and these results are generalizable for settings with similar economics and social characteristics. Also, the requested fee of €525.07 per month per pregnant woman is a strength of this study because of the applicability and thoughtfulness of this item. It is very likely that this price will actually cover the costs of a remote monitoring prenatal follow-up program. Finally, by adding this supplement to the RIZIV costs, there will be no increase in costs between the remote monitoring group and conventional care group in the three domains, but the prenatal follow-up and gestational outcomes will be improved for the remote monitoring group as we reported previously [10].

The main limitation of this study is its retrospective structure and the fact that the patients from the PREMOM study were not randomized. Nevertheless, the populations in the two arms were almost homogeneous regarding the baseline clinical characteristics. Second, the PREMOM study and this financial analysis provides a picture of real-life practice in Belgium; we did receive the data from the patient files and the hospital bills, but we do not have information of the patients’ hospital and medical consumption, or the patients’ social costs (eg, transportation and travel costs and the cost of lost employment income for the time spent for in hospital visits). Our results could also differ in different HCSs and different economic and social settings, such as in other countries. Additionally, this study is limited to 6 weeks after delivery. It is generally known that neonates that need intensive care at the time of their delivery will have a higher impact on health care costs then neonates who do not need this care. These costs are mostly due to rehospitalizations, acute care visits, or further intensive care for the rest of the infant’s life [15-19]. Further, we did not investigate the quality-adjusted life years (QALYs), which can be used as a generic measure of effectiveness. QALYs are a generic measure of disease burden, including both the quality and the quantity of the life lived, and it assesses the value of costs of medical interventions. To conclude, we evaluated only one type of remote monitoring follow-up program, which does not allow our results to be transferred to other proprietary technologies with varying transmission frequencies and methods of alert notifications.

### Comparisons With Previous Trials

Only two studies are known to have performed a cost analysis of a remote monitoring follow-up program in women with high-risk pregnancies. Morrison et al [9] performed a cost-effectiveness evaluation of remote monitoring in patients diagnosed with preterm labor. An average reduced cost of US $14,459 per pregnancy using remote monitoring services was obtained when compared to usual care. This cost reduction was due to reduced costs in antepartum hospitalization and intensive care nursery [9]. The conclusions of this article are in line with our main findings. Also, the study of Buysse et al [11] matches our principal findings. They obtained a cost reduction of €145,882 per year for high-risk pregnancies. But, unlike our study, these researchers did not use real-life data from patients in a remote monitoring program: they made a simulation exercise for all high-risk pregnancies that would qualify for home monitoring.

### Possible Explanations

The main objective of our study was to compare direct costs of a prenatal follow-up program for women diagnosed with GHD between remote monitoring and conventional care in hospital visits for a single-center population based on the initial assumption that remote monitoring technologies were provided with no additional costs. Early detection of clinical and device-related critical events provided by remote monitoring may have a positive impact on complication rates such as the development of severe hypertension, the need of inductions, prenatal hospitalizations, and neonatal hospitalizations. In our previously mentioned study, we reported a reduction in the prevalence of preeclampsia, hospitalization of the mother and the neonate, and inductions of labor [10]. In summary, by adding remote monitoring to the prenatal care of women at risk of these disorders, the risk of development of a severe hypertensive disorder is reduced and there are large potential benefits in terms of social and hospital expenditure restraint. These results can be read in Multimedia Appendix 5. In line with these benefits that are obtained with remote monitoring, the costs necessary for the medical care of the previously mentioned complications are reduced and/or avoided in the remote monitoring group and not in the conventional care group. The slightly higher costs of the medications for the patients of the remote monitoring group, when compared to conventional care group, can be explained by the higher need of medication for those patients. During the remote monitoring process, it is easy to make some changes in the antihypertensive treatment because their daily parameters are constantly at hand [10]. Women in the conventional care group will have less medication changes due to the lack of daily follow-up of their blood pressure.

The suggested €525.07 per month per pregnant woman fee for funding remote monitoring allows for HCS to not be elevated. By showing that there is no significant difference in costs between the remote monitoring group and conventional care group, a door is opened for policy makers charged with deciding how limited health care resources should be allocated in the era of exploding needs. This study, together with our previous report, states that better prenatal follow-up and gestational outcomes for the same cost as conventional care are possible by adding remote monitoring to the care of pregnant women with GHD.

### Recommendations for Further Research

Firstly, it would also be useful to investigate the QALYs for both the mother and the neonate who received remote monitoring to make further recommendations about this topic. This study is also shortened to postnatal follow-up until 6 weeks after delivery. It would be interesting to monitor the neonates in both groups—remote monitoring and conventional care groups—for longer than 6 weeks postpartum to get insights into the long-term cost benefits. Lastly, because the social costs (eg, transportation and travel costs and the cost of lost employment income for the time spent for in hospital visits) are not taken into account, it would be interesting to make additional analyses with these type of costs included. It is plausible that the differences in costs will be even greater when the previously mentioned items are taken into account.

### Conclusions

The results of this study show that a remote monitoring prenatal follow-up of women with GHD will not increase the costs for the HCS, RIZIV, or patient in comparison with conventional care. Furthermore, a RIZIV fee of €525.07 per month per pregnant woman allows the implementation of remote monitoring without increasing the health care costs for the remote monitoring group. These results are useful for policy makers charged with deciding how limited health care resources should be allocated in the era of exploding need. Further research of the long-term cost-effectiveness of remote monitoring, the QALYs, and social costs is recommended.

## Appendix

Multimedia Appendix 1 A detailed overview of the included costs.

Multimedia Appendix 2 The influence of the maternal demographics and characteristics on the health care costs.

Multimedia Appendix 3 Health care costs.

Multimedia Appendix 4 Detailed information the mean time of prenatal RM follow-up/pregnant woman.

Multimedia Appendix 5 Remote monitoring of hypertension diseases in pregnancy: a pilot study.

## Abbreviations

RM: remote monitoring
CC: conventional care
GHD: gestational hypertensive diseases
HCS: health care system
IQR: interquartile range
PREMOM study: Pregnancy Remote Monitoring study
QALY: quality-adjusted life years
RIZIV: National Institution for Insurance of Disease and Disability [Rijksinstituut voor Ziekte- en Invaliditeitverzekering]
