# Supplementary material for: Prenatal Remote Monitoring of Women With Gestational Hypertensive Diseases: Cost Analysis
Source: J Med Internet Res. 2018 Mar 26;20(3):e102. doi: 10.2196/jmir.9552 (PMC5891672; doi:10.2196/jmir.9552)
Supplement: Multimedia Appendix 1 [file jmir_v20i3e102_app1.pdf]

<u>Supplementary file 1:</u> A detailed overview of the included costs

1. Prenatal follow-up

- Cardiotocographics
- Prenatal visits
- Ultrasounds

2. Prenatal admission to the hospital

Costs related to the labs of the mother:

- Activated partial thromboplastin time
- Aerobic culture of sanies
- Counting of erythrocytes and/or hematocrit
- Counting of the leukocytes
- Counting of the thrombocytes
- Dosing of albumin in micro-amount by an immunological method
- Dosing of albumin
- Dosing of aspartate aminotransferase and alanine aminotransferase
- Dosing of calcium
- Dosing of chloride
- Dosing of creatinine
- Dosing of CRP with an immunological method
- Dosing of fibrinogen
- Dosing of glucose
- Dosing of glucose or other reducing sugars
- Dosing of hemoglobin by electrometric method
- Dosing of lactic dehydrogenases
- Dosing of magnesium
- Dosing of phosphates
- Dosing of potassium
- Dosing of sodium
- Dosing of total bilirubin and its fractions
- Dosing of total protein
- Dosing of total proteins
- Dosing of urea
- Dosing of uric acid
- Microscopic examination of urine sediment, after double staining
- Thromboplastin time

Costs of the medications:

- Pharmaceutical costs:
    - Aldomet® 250 mg
    - Amlor® 5 mg
    - Amlor® 10 mg
    - Atropine sulfate aguettant injection 0.5 mg/ml
    - Boostrix Polio® vaccine 0.5 ml
    - Bridion® injection 2 ml/100 mg
    - Buscopan® injection 1ml/20mg
    - Cabergoline Teva® 0.5 mg
    - Cedium Chlorhexidini® 0.0%
    - Cedium Chlorhexidini® 0.5%
    - Cafazoline Mylan® injection 2g
    - Celestone® injection 4 mg/ml
    - Chloramphenicol® 5 mg/ml
    - Clexane® SC injection 0.4 mg
    - Clexane® SC injection 0.6 mg

- Clindamycine Fresenius Kabi® 150 mg/ml
- Contramal® injection 100 mg/ml
- Cytotec® 200 mcg
- Dafalgan® 500 mg
- Daktarin® Spray 100g
- Diclofenac® suppo 100 mg
- Diprivan® injection 200 mg/20ml
- Edium Chlorhexidini® 0.05%
- Ephedrine® injection 50 mg/1ml
- Esmeron® injection 5 ml/50mg
- Fentanyl® injection 2 ml
- Fortal® 50 mg
- Glucuse injection 10 ml
- Glucose 5% infusion 100 ml
- Glucose 5% infusion 500 ml
- Glucose 5% infusion 1000 ml
- Hacdil - S® dilution 15 ml
- Hirudoid® gel
- Injectafer® 100mg/2ml
- Instillagel®
- Iso-Betadine® derm. 125 ml
- Iso-Betadine® gyn. 500 ml
- Iso-Betadine® hdryo-alkohol 500 ml
- Iso-Betadine® unigyn. 500 ml
- Linisol® 1% injection 10 ml
- Linisol® 2% injection 10 ml
- Litican® injection 50 mg/2ml
- Magnesium sulphate 1g/10ml
- Marcaine® injection 0.5%
- Movical Neutral®
- NaCl 0.9% 20 ml
- NaCl 0.9% perfusion Viaflo
- NaCl 0.9% perfusion 100 ml
- NaCl 0.9% perfusion 250 ml
- NaCl 0.9% perfusion 500 ml
- NaCl 0.9% perfusion 1000 ml
- Naropin® injection 20 ml 10mg/ml
- Neobacitracine® Pro instant
- Nepresol® 25 mg
- Norgalax® 120 mg
- Ondansetron Mylan® injection 2 mg
- Otrivine anti - rhinitis®
- Pabal® injection 1 ml/100mcg
- Paracetamol Actavis® perfusion 500 mg
- Paracetamol Fresenius Kabi® perfusion 10 mg/ml
- Paraffine 10 ml
- Penicilline 2.000.000E perfusion
- Phenylephrine® injection 50 mcg/1 ml
- Plasmalyte® 148 + Glucose 5%
- Plasmalyte® a viaflo 1000 ml

- Prepidil gel® 0.5 mg/3 ml
    - Primperan® injection 10 mg/2 ml
    - Prostin E2 comprime 0.5 mg
    - Reparil® 1% gel
    - Rhogam® injection 300 mcg
    - Riopan® gel 10 ml
    - Robinul + Neostigmine® injection 0.5 mg/ml
    - Ropivacaine Fresenius Kabi® injection 7.5 mg/ml
    - Scandicaïne® injection 1% 20 ml
    - Sufenta® injection 0.01 mg
    - Sufenta® injection 0.05 mg
    - Syntocinon® injection 10E/2 ml
    - Taradyl® injection 10 mg
    - Tardyferon® 80 mg
    - Trandate® 100 mg
    - Ultiva® injection 5 mg
    - Ultraproct® ointment 30 g
    - Vaseline® ointment 20g
    - Volulyte® 6% perfusion 500 ml
    - Xylocaine® + Adrenaline 10mg/ml + 5ug/ml
    - Zantac® 150 mg
- Patients costs for pharmaceutical products

Costs related to the admission:

- Cardiotocographics before the delivery
- Hospital care per admission
- Hospital care per day
- Medical imaging radiology
- Patients costs for clinical biology per day
- Personal share
- Supplement single room
- X-ray diagnosis

3. Maternal and neonatal care at and after delivery

Costs of the delivery:

- Additional fee for benefits with relative value
- Anesthesia
- Anesthesia for obstetric benefits
- Assistance provided by a physician in a hospital environment
- Delivery by cesarean section
- Delivery done by the midwife
- Monitoring and registration of fetal heart rate
- Normal or complicated delivery

Costs necessary for the care of the neonate:

- Activated partial thromboplastin time
- Aerobic culture of sanies
- Aortic puncture for decrease (s), injections, catheter insertion, etc.
- Biodimensional ultrasound
- Clinical examination of the newborn on the maternity
- Complicated dermatological correlation for extensive lesions, during hospitalization
- Counting of erythrocytes and/or hematocrit
- Counting of the leukocytes
- Counting of the thrombocytes
- Delivery margin of implants
- Determine anti-erythrocytes antibodies
- Determine blood groups
- Determine RH phenotype
- Dosing of albumin in micro-amount by an immunological method
- Dosing of ionized calcium outside each calculation method
- Dosing of sodium, potassium, chlorides and bicarbonates in plasma or serum
- Dosing of albumin
- Dosing of aspartate aminotransferase and alanine aminotransferase
- Dosing of calcium
- Dosing of chloride
- Dosing of creatinine
- Dosing of CRP with an immunological method
- Dosing of fibrinogen
- Dosing of glucose
- Dosing of glucose or other reducing sugars
- Dosing of hemoglobin by electrometric method
- Dosing of lactic dehydrogenases
- Dosing of magnesium
- Dosing of phosphates
- Dosing of potassium
- Dosing of sodium
- Dosing of total bilirubin and its fractions
- Dosing of total protein
- Dosing of total proteins
- Dosing of urea
- Dosing of uric acid
- Full blood and labile blood products - Fresh frozen human plasma virus inactivated

- Full transthoracic echographic bilan of the heart
- Hemoculture with identification of the isolated germs
- Hospital care per admission to the neonatal intensive care
- Hospital care per day at the neonatal intensive care
- Individual kinesiotherapy session where the personal involvement of the physiotherapist per beneficiary has a global average duration of 15 minutes
- Installation and monitoring of positive pressure ventilation by nasal route using probe or mask and artificial respiratory equipment
- Installation and supervision of controlled or assisted continuous ventilation
- Installation and supervision on the continues monitoring of the heart function of the neonate older than 33 weeks
- Intravenous perfusion to child younger than seven years old
- Larynx intubation
- Medical imaging radiology
- Microscopic examination of urine sediment, after double staining
- Patients costs for clinical biology per day
- Patients costs for the admission to the intensive care
- Peripherally inserted central venous catheter (PICC) for long-term use
- Personal share for the admission to the neonatal intensive care
- pH determination and CO2- and O2 pressures in the blood (acid base equilibrium)
- Placement of an umbilical catheter in the newborn outside the anesthesia
- Stomach catheterization in children less than seven years
- Surveillance from day 6 until day 12 after the delivery on the maternity
- Take charge of newborns by at risk pregnancies
- Thromboplastin time
- Total abdominal investigation with at least eight incisions
- X-ray diagnosis

Other costs:

- Admission to the emergency room
- Admission to the intensive care
- Blood derives
- Patients costs for the admission to the intensive care
