# Supplementary material for: Prenatal Remote Monitoring of Women With Gestational Hypertensive Diseases: Cost Analysis
Source: J Med Internet Res. 2018 Mar 26;20(3):e102. doi: 10.2196/jmir.9552 (PMC5891672; doi:10.2196/jmir.9552)
Supplement: Multimedia Appendix 2 [file jmir_v20i3e102_app2.pdf]

Supplementary file 2: Multivariable model for the prediction of healthcare costs using maternal demographics and characteristics

Variable	*Beta*	*95.0% CI for B*		*P-value*
		Lower Bound	*Upper Bound*	
**Outcome 1:** HCS costs of prenatal visits				
RM vs. no RM	-1.20	-42.65	13.59	0.31
Maternal age, 1 year increase	-0.02	-3.05	2.43	0.82
Pre-pregnancy weight, 1 kg increase	-0.35	-5.38	2.84	0.54
Height, 1 cm increase	0.21	-2.33	6.46	0.35
BMI, 1 kg/m² increase	0.33	-7.93	14.96	0.54
Primigravida vs. multigravida	-0.14	-46.77	7.78	0.16
Smoking vs. no smoking	-0.00	-60.12	59.04	0.99
**Outcome 2:** RIZIV costs of prenatal visits				
RM vs. no RM	-1.20	-25.59	8.16	0.31
Maternal age, 1 year increase	-0.02	-1.83	1.46	0.82
Pre-pregnancy weight, 1 kg increase	-0.35	-3.23	1.71	0.54
Height, 1 cm increase	0.21	-1.40	3.88	0.35
BMI, 1 kg/m² increase	0.33	-4.76	8.98	0.54
Primigravida vs. multigravida	-0.14	-28.07	4.67	0.16
Smoking vs. no smoking	-0.00	-36.08	35.43	0.99
**Outcome 3:** patients costs for prenatal visits				
RM vs. no RM	-1.20	-18.05	5.43	0.31
Maternal age, 1 year increase	-0.02	-1.22	0.97	0.82
Pre-pregnancy weight, 1 kg increase	-0.35	-2.15	1.14	0.54
Height, 1 cm increase	0.21	-0.93	2.58	0.35
BMI, 1 kg/m² increase	0.33	-3.17	5.98	0.54
Primigravida vs. multigravida	-0.14	-18.70	3.11	0.16
Smoking vs. no smoking	-0.00	-24.04	23.61	0.99
**Outcome 4:** HCS costs for ultrasounds				
RM vs. no RM	-0.07	-31.59	15.02	0.48
Maternal age, 1 year increase	0.11	-0.99	3.55	0.27
Pre-pregnancy weight, 1 kg increase	-0.48	-4.86	1.96	0.40
Height, 1 cm increase	0.20	-1.98	5.30	0.37
BMI, 1 kg/m² increase	0.40	-5.86	13.10	0.45
Primigravida vs. multigravida	-0.00	-22.74	22.47	0.99
Smoking vs. no smoking	0.06	-34.46	64.30	0.55
**Outcome 5:** RIZIV costs for ultrasounds				
RM vs. no RM	-0.07	-28.65	13.62	0.48
Maternal age, 1 year increase	0.11	-0.90	3.22	0.27
Pre-pregnancy weight, 1 kg increase	-0.48	-4.41	1.77	0.40
Height, 1 cm increase	0.20	-1.80	4.80	0.37
BMI, 1 kg/m² increase	0.40	-5.33	11.87	0.45
Primigravida vs. multigravida	-0.00	-20.62	20.38	0.99
Smoking vs. no smoking	0.06	-31.25	58.30	0.55
**Outcome 6:** patients costs for ultrasounds				
RM vs. no RM	-0.07	-2.95	1.40	0.48
Maternal age, 1 year increase	0.11	-0.09	0.33	0.27
Pre-pregnancy weight, 1 kg increase	-0.48	-0.45	0.18	0.40
Height, 1 cm increase	0.20	-0.19	0.50	0.37
BMI, 1 kg/m² increase	0.40	-0.55	1.22	0.45
Primigravida vs. multigravida	-0.00	-2.12	2.10	0.99
Smoking vs. no smoking	0.06	-3.21	6.00	0.55
**Outcome 7:** HCS costs for cardiotocographics				
RM vs. no RM	0.12	-17.53	76.34	0.22
Maternal age, 1 year increase	-0.02	-5.12	4.02	0.81
Pre-pregnancy weight, 1 kg increase	0.15	-5.96	7.76	0.80

Height, 1 cm increase	-0.04	-8.02	6.65	0.85
BMI, 1 kg/m² increase	-0.03	-19.71	18.50	0.95
Primigravida vs. multigravida	-0.12	-74.24	16.83	0.21
Smoking vs. no smoking	0.02	-88.27	110.63	0.82
**Outcome 8:** RIZIV costs for cardiotocographics				
RM vs. no RM	0.12	-8.77	38.17	0.22
Maternal age, 1 year increase	-0.02	-2.56	2.01	0.81
Pre-pregnancy weight, 1 kg increase	0.15	-2.98	3.88	0.80
Height, 1 cm increase	-0.04	-4.01	3.33	0.85
BMI, 1 kg/m² increase	-0.03	-9.85	9.25	0.95
Primigravida vs. multigravida	-0.12	-37.12	8.42	0.21
Smoking vs. no smoking	0.02	-44.13	55.31	0.82
**Outcome 9:** patients costs for cardiotocographics				
RM vs. no RM	0.12	-8.77	38.17	0.22
Maternal age, 1 year increase	-0.02	-2.56	2.01	0.81
Pre-pregnancy weight, 1 kg increase	0.15	-2.98	3.88	0.80
Height, 1 cm increase	-0.04	-4.01	3.33	0.85
BMI, 1 kg/m² increase	-0.03	-9.85	9.25	0.95
Primigravida vs. multigravida	-0.12	-37.12	8.42	0.21
Smoking vs. no smoking	0.02	-44.13	55.32	0.82
**Outcome 10:** HCS costs for labs				
RM vs. no RM	-0.07	-28.08	12.79	0.46
Maternal age, 1 year increase	-0.07	-2.71	1.26	0.47
Pre-pregnancy weight, 1 kg increase	0.22	-2.44	3.53	0.72
Height, 1 cm increase	-0.06	-3.65	2.74	0.78
BMI, 1 kg/m² increase	-0.09	-9.05	7.59	0.86
Primigravida vs. multigravida	0.15	-4.19	35.46	0.12
Smoking vs. no smoking	0.03	-37.08	49.51	0.78
**Outcome 11:** RIZIV costs for labs				
RM vs. no RM	-0.07	-28.07	8.67	0.30
Maternal age, 1 year increase	-0.07	-2.49	1.08	0.44
Pre-pregnancy weight, 1 kg increase	0.22	-2.15	3.22	0.69
Height, 1 cm increase	-0.07	-3.34	2.40	0.75
BMI, 1 kg/m² increase	-0.11	-8.30	6.65	0.83
Primigravida vs. multigravida	0.19	-0.20	35.45	0.05
Smoking vs. no smoking	-0.01	-40.89	36.95	0.92
**Outcome 12:** patients costs for labs				
RM vs. no RM	0.09	-2.59	6.70	0.38
Maternal age, 1 year increase	-0.01	-0.47	0.43	0.93
Pre-pregnancy weight, 1 kg increase	0.01	-0.67	0.69	0.98
Height, 1 cm increase	0.01	-0.70	0.74	0.96
BMI, 1 kg/m² increase	0.05	-1.80	1.98	0.92
Primigravida vs. multigravida	-0.09	-6.49	2.51	0.38
Smoking vs. no smoking	0.16	-1.65	18.02	0.10
**Outcome 13:** HCS costs for prenatal admission				
RM vs. no RM	0.13	-113.21	567.20	0.19
Maternal age, 1 year increase	0.04	-25.29	40.94	0.64
Pre-pregnancy weight, 1 kg increase	0.09	-45.91	53.58	0.88
Height, 1 cm increase	-0.07	-61.98	44.35	0.74
BMI, 1 kg/m² increase	-0.11	-153.52	123.40	0.83
Primigravida vs. multigravida	0.23	60.34	720.41	**0.02**
Smoking vs. no smoking	-0.01	-749.96	691.68	0.94
**Outcome 14:** RIZIV costs for prenatal admission				
RM vs. no RM	0.09	-98.26	267.62	0.36
Maternal age, 1 year increase	0.03	-15.35	20.26	0.79
Pre-pregnancy weight, 1 kg increase	0.20	-22.10	31.40	0.73

Height, 1 cm increase	-0.11	-35.64	21.54	0.63
BMI, 1 kg/m² increase	-0.18	-87.10	61.81	0.74
Primigravida vs. multigravida	0.23	34.51	389.45	**0.02**
Smoking vs. no smoking	-0.03	-439.18	336.04	0.79
**Outcome 15:** patients costs for prenatal admission				
RM vs. no RM	0.16	-35.59	320.22	0.12
Maternal age, 1 year increase	0.06	-11.95	22.69	0.54
Pre-pregnancy weight, 1 kg increase	-0.04	-26.82	25.20	0.95
Height, 1 cm increase	-0.03	-29.57	26.04	0.90
BMI, 1 kg/m² increase	-0.04	-74.82	69.99	0.95
Primigravida vs. multigravida	0.20	5.81	350.98	**0.04**
Smoking vs. no smoking	0.01	-354.50	399.37	0.91
**Outcome 16:** HCS costs for medicaments				
RM vs. no RM	-0.01	-42.48	39.23	0.94
Maternal age, 1 year increase	-0.08	-5.60	2.36	0.42
Pre-pregnancy weight, 1 kg increase	0.26	-4.56	7.39	0.64
Height, 1 cm increase	-0.20	-9.36	3.41	0.36
BMI, 1 kg/m² increase	-0.23	-20.37	12.88	0.66
Primigravida vs. multigravida	0.25	12.59	91.85	**0.01**
Smoking vs. no smoking	0.00	-85.19	87.83	0.98
**Outcome 17:** RIZIV costs for medicaments				
RM vs. no RM	0.03	-20.55	26.87	0.79
Maternal age, 1 year increase	-0.11	-3.62	0.99	0.26
Pre-pregnancy weight, 1 kg increase	0.31	-2.53	4.41	0.59
Height, 1 cm increase	-0.15	-4.94	2.47	0.51
BMI, 1 kg/m² increase	-0.24	-11.82	7.48	0.66
Primigravida vs. multigravida	0.14	-6.33	39.68	0.15
Smoking vs. no smoking	-0.02	-54.05	46.43	0.88
**Outcome 18:** patients costs for medicaments				
RM vs. no RM	-0.07	-34.64	16.25	0.48
Maternal age, 1 year increase	-0.01	-2.60	2.35	0.92
Pre-pregnancy weight, 1 kg increase	0.27	-2.80	4.64	0.63
Height, 1 cm increase	-0.17	-5.52	2.43	0.44
BMI, 1 kg/m² increase	-0.31	-13.51	7.20	0.55
Primigravida vs. multigravida	0.29	12.84	62.20	**0.003**
Smoking vs. no smoking	0.01	-50.05	57.76	0.89
**Outcome 19:** HCS costs for delivery				
RM vs. no RM	0.05	-146.18	240.89	0.63
Maternal age, 1 year increase	0.03	-15.54	22.14	0.73
Pre-pregnancy weight, 1 kg increase	0.11	-25.52	31.07	0.85
Height, 1 cm increase	-0.21	-45.21	15.28	0.33
BMI, 1 kg/m² increase	-0.28	-100.44	57.10	0.59
Primigravida vs. multigravida	0.10	-94.59	280.91	0.33
Smoking vs. no smoking	-0.03	-476.50	343.61	0.75
**Outcome 20:** RIZIV costs for delivery				
RM vs. no RM	-0.03	-88.19	63.87	0.75
Maternal age, 1 year increase	0.09	-3.91	10.89	0.35
Pre-pregnancy weight, 1 kg increase	0.22	-8.95	13.29	0.70
Height, 1 cm increase	-0.30	-20.16	3.61	0.17
BMI, 1 kg/m² increase	-0.26	-38.70	23.19	0.62
Primigravida vs. multigravida	0.13	-23.79	123.73	0.18
Smoking vs. no smoking	0.08	-95.02	227.17	0.42
**Outcome 21:** patients costs for delivery				
RM vs. no RM	0.07	-98.54	217.57	0.46
Maternal age, 1 year increase	-0.00	-15.57	15.20	0.98
Pre-pregnancy weight, 1 kg increase	0.03	-22.51	23.71	0.96

Height, 1 cm increase	-0.12	-31.39	18.01	0.59
BMI, 1 kg/m² increase	-0.22	-78.24	50.41	0.70
Primigravida vs. multigravida	0.05	-110.14	196.52	0.58
Smoking vs. no smoking	-0.07	-467.40	202.36	0.43
**Outcome 22:** HCS costs for neonatal care				
RM vs. no RM	-0.08	-2117.84	936.95	0.45
Maternal age, 1 year increase	-0.01	-158.39	138.95	0.90
Pre-pregnancy weight, 1 kg increase	-0.33	-287.56	159.07	0.57
Height, 1 cm increase	0.01	-232.67	244.74	0.96
BMI, 1 kg/m² increase	0.31	-438.78	804.51	0.56
Primigravida vs. multigravida	0.11	-690.95	2272.51	0.29
Smoking vs. no smoking	-0.03	-3789.20	2683.23	0.74
**Outcome 23:** RIZIV costs for neonatal care				
RM vs. no RM	-0.07	-1960.64	895.27	0.46
Maternal age, 1 year increase	-0.01	-145.37	132.60	0.93
Pre-pregnancy weight, 1 kg increase	-0.35	-272.74	144.82	0.55
Height, 1 cm increase	0.01	-216.34	229.99	0.95
BMI, 1 kg/m² increase	0.34	-394.42	769.92	0.52
Primigravida vs. multigravida	0.11	-637.58	2132.94	0.29
Smoking vs. no smoking	-0.03	-3515.15	2535.86	0.75
**Outcome 24:** patients costs for neonatal care				
RM vs. no RM	-0.09	-182.45	66.94	0.36
Maternal age, 1 year increase	-0.05	-15.47	8.80	0.59
Pre-pregnancy weight, 1 kg increase	-0.02	-18.52	17.95	0.98
Height, 1 cm increase	-0.02	-20.28	18.70	0.94
BMI, 1 kg/m² increase	-0.12	-56.63	44.87	0.82
Primigravida vs. multigravida	0.07	-77.86	164.07	0.48
Smoking vs. no smoking	-0.05	-327.54	200.87	0.64
**Outcome 25:** HCS costs for other				
RM vs. no RM	-0.14	-95.75	17.13	0.17
Maternal age, 1 year increase	-0.13	-9.32	1.67	0.17
Pre-pregnancy weight, 1 kg increase	-0.15	-9.35	7.16	0.79
Height, 1 cm increase	0.22	-4.35	13.29	0.32
BMI, 1 kg/m² increase	0.10	-20.72	25.22	0.85
Primigravida vs. multigravida	0.03	-45.68	63.82	0.74
Smoking vs. no smoking	-0.09	-174.62	64.53	0.36
**Outcome 26:** RIZIV costs for other				
RM vs. no RM	-0.14	-96.04	17.21	0.17
Maternal age, 1 year increase	-0.12	-9.21	1.81	0.19
Pre-pregnancy weight, 1 kg increase	-0.14	-9.30	7.26	0.81
Height, 1 cm increase	0.21	-4.53	13.17	0.34
BMI, 1 kg/m² increase	0.09	-21.00	25.10	0.86
Primigravida vs. multigravida	0.04	-43.69	66.18	0.69
Smoking vs. no smoking	-0.09	-174.48	65.47	0.37
**Outcome 27:** patients costs for other				
RM vs. no RM	0.00	-5.11	5.32	0.97
Maternal age, 1 year increase	-0.05	-0.63	0.38	0.63
Pre-pregnancy weight, 1 kg increase	-0.11	-0.84	0.39	0.85
Height, 1 cm increase	0.09	0.70	0.97	0.70
BMI, 1 kg/m² increase	0.10	0.85	2.33	0.85
Primigravida vs. multigravida	-0.09	0.40	2.89	0.40
Smoking vs. no smoking	-0.01	0.92	10.51	0.92
**Outcome 28:** HCS costs for total amount of costs				
RM vs. no RM	-0.04	-1951.04	1300.87	0.69
Maternal age, 1 year increase	-0.01	-162.26	154.27	0.96
Pre-pregnancy weight, 1 kg increase	-0.28	-296.41	179.05	0.63

Height, 1 cm increase	-0.02	-268.21	240.00	0.91
BMI, 1 kg/m² increase	0.24	-512.57	810.84	0.66
Primigravida vs. multigravida	0.16	-290.22	2864.47	0.11
Smoking vs. no smoking	-0.04	-4117.46	2771.61	0.70
**Outcome 29:** RIZIV costs for total amount of costs				
RM vs. no RM	-0.07	-2010.89	976.01	0.49
Maternal age, 1 year increase	-0.01	-152.65	138.08	0.92
Pre-pregnancy weight, 1 kg increase	-0.31	-278.14	158.57	0.59
Height, 1 cm increase	-0.00	-235.19	231.61	0.99
BMI, 1 kg/m² increase	-0.31	-431.23	784.42	0.57
Primigravida vs. multigravida	0.14	-417.26	2480.34	0.16
Smoking vs. no smoking	-0.03	-3680.48	2648.09	0.75
**Outcome 30:** patients costs for total amount of costs				
RM vs. no RM	0.09	-161.81	458.05	0.35
Maternal age, 1 year increase	0.01	-29.29	31.04	0.95
Pre-pregnancy weight, 1 kg increase	0.01	-44.83	45.80	0.98
Height, 1 cm increase	-0.10	-59.26	37.61	0.66
BMI, 1 kg/m² increase	-0.19	-149.13	103.16	0.72
Primigravida vs. multigravida	0.18	-19.94	581.39	0.07
Smoking vs. no smoking	-0.05	-816.55	496.81	0.63
CI = Confidence interval, RM = remote monitoring				
