# Supplementary material for: Prenatal Remote Monitoring of Women With Gestational Hypertensive Diseases: Cost Analysis
Source: J Med Internet Res. 2018 Mar 26;20(3):e102. doi: 10.2196/jmir.9552 (PMC5891672; doi:10.2196/jmir.9552)
Supplement: Multimedia Appendix 3 [file jmir_v20i3e102_app3.pdf]

**Supplementary file 3:** Healthcare costs						
		**Study group**		**Cost saving in the RM group**		**Statistical significance (2 – tailed)**
		RM group (n = 43)	*CC group (n = 97)*	€	%	
Total amount of costs						
- HCS costs (€)	Mean	4233.31 (± 3463.31)	4973.69 (± 5219.00)	740.38	14.89	0.82
	Median	3317.97 (2832.16 – 3910.34)	3287.98 (2708.22 – 4542.21)			
- RIZIV costs (€)	Mean	2797.42 (± 2905.18)	3646.39 (± 4878.47	848.97	23.18	0.19
	Median	1904.68 (1747.65 – 2399.10)	2304.52 (1729.86 – 2872.81)			
- Patients costs (€)	Mean	1435.89 (± 829.09)	1327.30 (± 753.94)	-108.59	-8.18	0.38
	Median	1332.95 (857.32 – 1750.41)	1270.58 (648.33 – 1738.51)			

Values are means ± SD and median with inter-quartile in euros (€); costs savings are calculated in euros (€) and percentages (%).
RM = remote monitoring; CC = conventional care; HCS = health care system; RIZIV = national healthcare insurances
