# Supplementary material for: Prenatal Remote Monitoring of Women With Gestational Hypertensive Diseases: Cost Analysis
Source: J Med Internet Res. 2018 Mar 26;20(3):e102. doi: 10.2196/jmir.9552 (PMC5891672; doi:10.2196/jmir.9552)
Supplement: Multimedia Appendix 5 [file jmir_v20i3e102_app5.pdf]

Original Paper

Remote Monitoring of Hypertension Diseases in Pregnancy: A Pilot Study

Dorien Lanssens[1,2], MSc; Thijs Vandenberk[1], MSc; Christophe JP Smeets[1], MSc; Hélène De Cannière[1], MSc; Geert Molenberghs[3], PhD; Anne Van Moerbeke[1], MSc; Anne van den Hoogen[1], MSc; Tiziana Robijns[1], MSc; Sharona Vonck[2], MSc; Anneleen Staelens[2], MD; Valerie Storms[1], PhD; Inge M Thijs[1,4], PhD; Lars Grieten[1], PhD; Wilfried Gyselaers[1,2,5], MD, PhD

[1]Mobile Health Unit, Faculty of Medicine and Life Sciences, Hasselt University, Hasselt, Belgium

[2]Department of Gynaecology, Ziekenhuis Oost Limburg, Genk, Belgium

[3]Interuniversity Institute for Biostatics and Statistical Bioinformatics, Hasselt University & KULeuven, Hasselt, Belgium

[4]Future Health Department, Ziekenhuis Oost-Limburg, Genk, Belgium

[5]Department of Physiology, Hasselt University, Hasselt, Belgium

Corresponding Author:
Dorien Lanssens, MSc
Mobile Health Unit
Faculty of Medicine and Life Sciences
Hasselt University
Martelarenlaan 42
Hasselt, 3500
Belgium
Phone: 32 0494919476
Fax: 32 89 32 50 50
Email: dorien.lanssens@uhasselt.be

Abstract

Background: Although remote monitoring (RM) has proven its added value in various health care domains, little is known about the remote follow-up of pregnant women diagnosed with a gestational hypertensive disorders (GHD).

Objective: The aim of this study was to evaluate the added value of a remote follow-up program for pregnant women diagnosed with GHD.

Methods: A 1-year retrospective study was performed in the outpatient clinic of a 2nd level prenatal center where pregnant women with GHD received RM or conventional care (CC). Primary study endpoints include number of prenatal visits and admissions to the prenatal observation ward. Secondary outcomes include gestational outcome, mode of delivery, neonatal outcome, and admission to neonatal intensive care (NIC). Differences in continuous and categorical variables in maternal demographics and characteristics were tested using Unpaired Student's two sampled *t* test or Mann-Whitney *U* test and the chi-square test. Both a univariate and multivariate analysis were performed for analyzing prenatal follow-up and gestational outcomes. All statistical analyses were done at nominal level, Cronbach alpha=.05.

Results: Of the 166 patients diagnosed with GHD, 53 received RM and 113 CC. After excluding 5 patients in the RM group and 15 in the CC group because of the missing data, 48 patients in RM group and 98 in CC group were taken into final analysis. The RM group had more women diagnosed with gestational hypertension, but less with preeclampsia when compared with CC (81.25% vs 42.86% and 14.58% vs 43.87%). Compared with CC, univariate analysis in RM showed less induction, more spontaneous labors, and less maternal and neonatal hospitalizations (48.98% vs 25.00%; 31.63% vs 60.42%; 74.49% vs 56.25%; and 27.55% vs 10.42%). This was also true in multivariate analysis, except for hospitalizations.

Conclusions: An RM follow-up of women with GHD is a promising tool in the prenatal care. It opens the perspectives to reverse the current evolution of antenatal interventions leading to more interventions and as such to ever increasing medicalized antenatal care.

(JMIR Mhealth Uhealth 2017;5(3):e25) doi:10.2196/mhealth.6552

XSL•FO
RenderX

KEYWORDS
pregnancy; gestational hypertension disorders; eHealth; remote monitoring

Introduction

Background

Gestational hypertensive disorders (GHD) remain one of the most significant and intriguing unsolved problems in obstetrics [1,2]. It is estimated that 5-10% of pregnancies are complicated by this disease, and it is one of the major causes of maternal and fetal morbidity and mortality [1,3,4]. GHD is defined as new onset hypertension (diastolic blood pressure ≥90 mmHg and systolic blood pressure ≥140 mmHg), with or without proteinuria (≥300 mg in 24-h urine collection) at or after 20 weeks of gestation [1]. The most common management for GHD in Belgium is an admission to the prenatal observation unit for diagnostic and therapeutic follow-up before induction of labor or discharge at home. In severe cases, premature birth is indicated [1].

As part of the Hasselt University and the Limburg Clinical Research Program (LCRP), Ziekenhuis Oost-Limburg (Genk, Belgium) initiated in January 2015 a remote monitoring (RM) program for women with or at risk for GHD. RM is an alternative approach in medical management (dating back to the early 1990s) facilitating patients' management at home [5]. It is defined as the use of telecommunication technologies to assist the transmission of medical information and services between health care providers and patients. The use of this 2-way telecommunication technology, using multimedia and computer networks, to assist medical management is a growing trend internationally [6].

In this paper, we report our first clinical results of RM in GHD, obtained retrospectively during the year of technical installation of remote communication between hospital doctors or midwives and pregnant women at home.

Related Work

RM has already shown benefits in Cardiology and Pneumology [7,8]. In the prenatal care, RM has also shown an added value to improve maternal and neonatal outcomes. Various studies reported a reduction in unscheduled patient visits, low neonatal birth weight, and admissions to neonatal intensive care (NIC) for pregnant women who received RM compared with pregnant women who did not receive these devices. Additionally, RM can contribute to significant reductions in health care costs. RM was also demonstrated to prolong gestational age and to improve feelings of self-efficacy, maternal satisfaction, and gestational age at delivery when compared with a control group which did not received RM [9-16]. Unfortunately, some of the previous mentioned studies are dating back to 1995 and no more recent work is available. This is in contradiction with the rapid technological advancements that have been seen in the last decade. Further, no studies are published about the added value of RM in pregnant women with GHD. To our knowledge, this is the first publication about a prenatal follow-up program for pregnant women with GHD to date.

Methods

Subjects

All women diagnosed with GHD who delivered at the outpatient prenatal clinic of Ziekenhuis Oost-Limburg (Genk, Belgium) during 2015 were included. Women received RM on demand of the responsible obstetrician before admission or after discharge from the prenatal observation ward. The criteria to initiate RM were GHD at gestational age ≥20 weeks where an intensive follow-up until delivery was desirable. Women without a mobile phone, a gestational age less than 20 weeks, a fetus with congenital malformations, and women who refused informed consent were excluded and received conventional care (CC).

Between January 1, 2015 and December 31, 2015, there were 2058 women who had prenatal care and delivery at Ziekenhuis Oost-Limburg. It was found that 166 women were diagnosed with GHD, 53 of them received CC added with RM. The remaining 113 pregnant women with GHD did not receive RM but only CC.

Interventions in the Remote Monitoring Group

Women consenting for RM received obstetric surveillance by a Withings Wireless Blood Pressure Monitor, Withings Smart Body Analyzer, and a Withings Pulse O² (Withings, Issy-les-Moulineux, France). Pregnant women participating in the prenatal remote follow-up program were asked to perform one blood pressure measurement in the morning and one in the evening, one weight measurement a day, and wear an activity tracker day and night until delivery or hospital admission (see Figure 1).

The data from the monitor devices were transmitted to a Web-based dashboard developed by the Mobile Health Unit of the University of Hasselt. Predetermined alarm signals were set; one midwife performed remote follow-up of all transformed data at the dashboard. She had to discriminate normal and alarm signals of systolic blood pressure >140 mmHg, diastolic blood pressure >90 mmHg, or weight gain >1 kg/day. Alarm events were communicated with the obstetrician in charge to discuss management options before contacting and instructing patients at home. Type of interventions were (1) expectant management, (2) ambulatory blood sampling and 24-h urine collection at home, (3) adjustment of the antihypertensive therapy or physical activity, (4) admission to the antenatal ward, and (5) induction of labor. Therapeutic interventions were according to local management.

The hospital's Medical Ethics Committee approved the study.

XSL•FO
RenderX

Figure 1. The equipment used in the remote monitoring group.

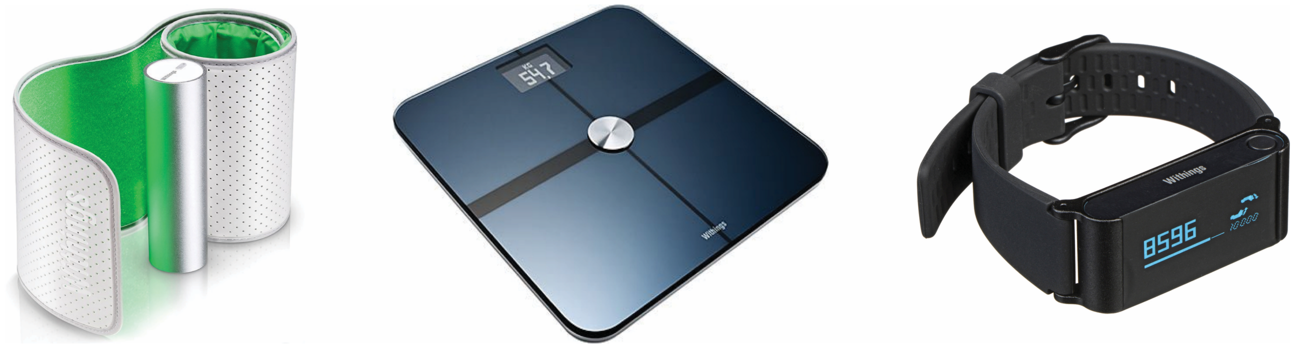

Maternal Demographics

Maternal demographics and characteristics of the patients in the RM group were collected at study entry. In the CC group, these data were obtained by manual search through the electronic medical records.

Primary Outcome: Prenatal Follow-Up

Total numbers of prenatal consultations were collected from 10 weeks of gestation onwards: ultrasound scans, cardiotocographics (CTG), admission to the prenatal ward, total days of hospitalization, and the number of admissions until delivery. These data were retrospectively collected from the electronic medical records after the delivery of the women in both the RM and CC group. These data were checked with the hospital administration and billing records.

Secondary Outcomes: Delivery Outcomes

Maternal parameters collected at birth were gestational age at delivery and mode of delivery. Neonatal outcomes collected were birth weight, birth weight percent, length, Apgar at 1′ and 5′, and number of admissions to NIC.

Statistical Analysis

Differences in continuous and categorical variables in maternal demographics and characteristics were tested using Unpaired Student’s two sampled *t* test or Mann-Whitney *U* test and the chi-square test. Both univariate and multivariate analyses were performed for analyzing prenatal follow-up and gestational outcomes. Beta coefficients and 95% CI were calculated for multivariate analysis. All statistical analyses are done at nominal level, Cronbach alpha=.05. Statistical analysis was performed with Statistical Package for Social Sciences release 22.0 (IBM SPSS Inc).

Results

Participant Demographics

Of the 2058 deliveries in Ziekenhuis Oost-Limburg in 2015, 18.06% (166/2058) were diagnosed with GHD and had both prenatal care and birth in the same hospital. A total of 31.92% (53/166) (31.92%) of the GHD pregnancies had RM. Of these, 3.01% (5/53) were excluded from analysis because of missing data (n=4) and fetal loss (n=1). In total, 28.92% (48/166) RM women were eligible for analysis. The other 68.08% (133/166) GHD pregnancies had CC. Of these, 9.04% (15/133) women were excluded because of missing data, leaving 59.04% (98/166) eligible for analysis. Figure 2 shows the study population in a flowchart.

Table 1 shows the maternal demographics and characteristics of the women diagnosed with GHD. In CC, there were more primigravidas and smokers than in RM: 66.32% (65/98) versus 41.66% (20/48) and 10.20% (10/98) versus 0% (0/48), respectively.

XSL•FO
RenderX

Table 1. Maternal demographics and characteristics.

Variable	RM[a] group (n=48)	CC[b] group (n=98)	Statistical significance (2-tailed), *P* value
Maternal age in years, mean (SD)	31.69 (4.25)	31.94 (4.77)	.73
Pre pregnancy weight (kg), mean (SD)	72.00 (17.99)	76.80 (19.74)	.11
Height (cm), mean (SD)	166.00 (6.94)	167.08 (6.86)	.38
BMI (kg/m²), mean (SD)	25.54 (5.58)	27.08 (6.92)	.32
Primigravidity, n (%)	20 (41.66)	65 (66.32)	.005
**Concomitant diseases, n (%)**			
Cardiovascular disorders	0 (0)	1 (1.02)	.48
Blood coagulation disorder	1 (2.08)	1 (1.02)	.61
Endocrine disorders	2 (4.16)	5 (5.10)	.81
Immunological disorders	1 (2.08)	2 (2.04)	.99
Smoking, n (%)	0 (0)	10 (10.20)	.02
GA[c] first visit in weeks, mean (SD)	10.10 (5.36)	11.21 (7.60)	.66

[a]RM: remote monitoring.
[b]CC: conventional care.
[c]GA: gestational age.

Figure 2. The study population.

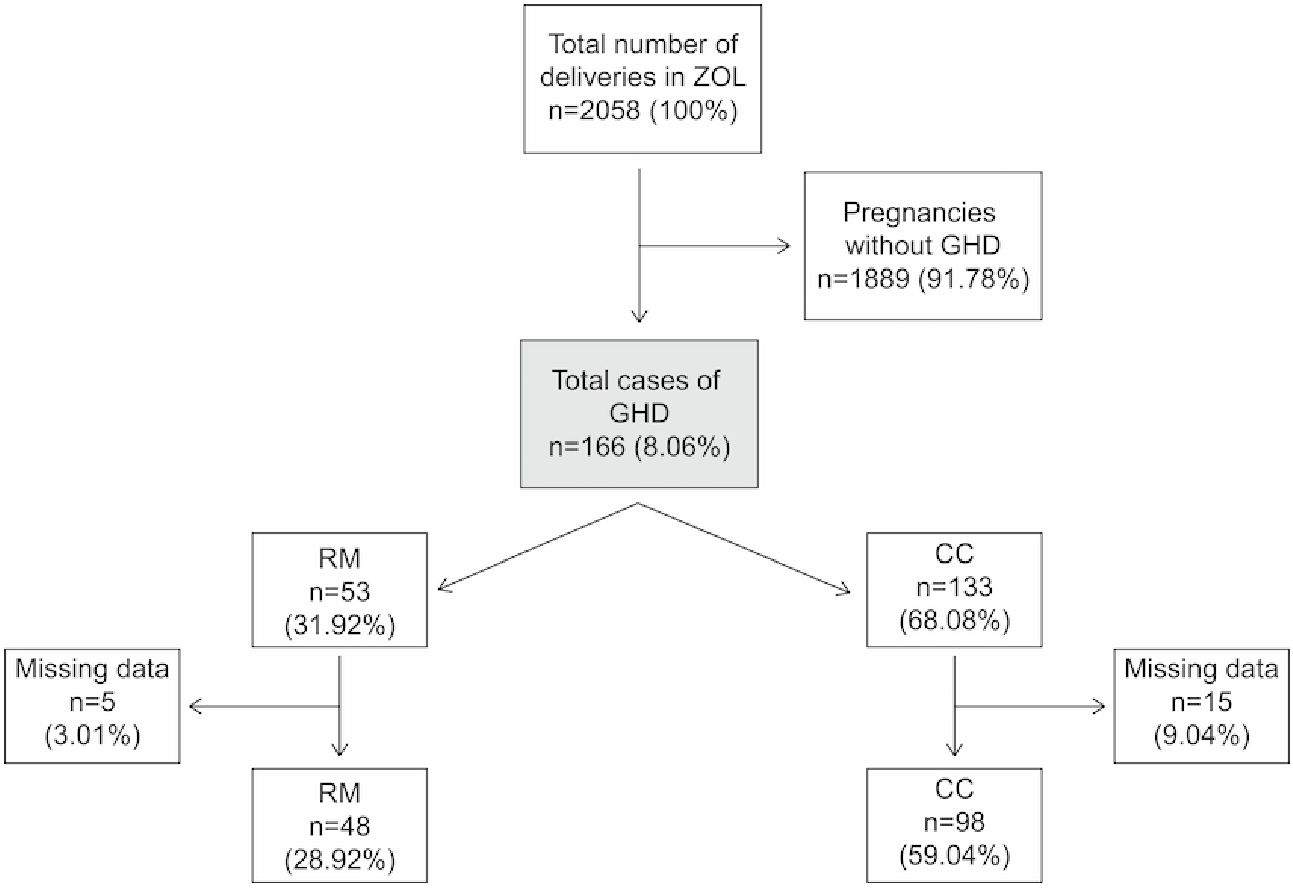

XSL•FO
RenderX

Prenatal Follow-Up: Comparison Between RM and CC

Data on prenatal follow-up balance are shown in Table 2. The number of prenatal hospital admissions and admissions until delivery were lower in RM than in CC when a univariate analysis is performed: 56.25% (27/48) versus 74.49% (73/98), and 27.08% (13/48) versus 62.24% (61/97). This was not significant in multivariate analysis. For both uni- and multivariate analysis was the prevalence of gestational hypertension higher in RM than in CC (81.25% vs 42.86% and beta=6.62), but the prevalence of preeclampsia was lower (14.85% vs 43.87% and beta=.24).

Table 2. Prenatal follow-up.

Variable	Univariate analysis			Multivariate analysis		
	RM[a] group (n=48)	CC[b] group (n=98)	*P* value	RM versus no RM (beta)	95% CI[c] for beta	*P* value
Total number of prenatal visits, mean (SD)	8.77 (4.12)	8.86 (3.51)	.90	−.56	−1.74 to 9.14	.54
CTG's, mean (SD)	2.23 (2.05)	1.89 (1.70)	.46	−.08	−1.12 to 0.53	.48
Echo's, mean (SD)	3.95 (2.00)	3.67 (2.12)	.08	.07	−0.56 to 1.19	.48
Prenatal admission, n (%)	27 (56.25)	73 (74.49)	.03	.46	0.18-1.45	.09
Days hospitalized, mean (SD)	5.74 (8.98)	4.73 (5.69)	.57	.10	−1.62 to 4.81	.32
Prenatal admission until delivery, n (%)	13 (27.08)	61 (62.24)	<.001	.38	0.12-1.22	.11
**Gestational outcome, n (%)**						
Essential hypertension	1 (2.08)	9 (9.18)	.11			
Gestational hypertension	39 (81.25)	42 (42.86)	<.001	6.62	2.40-18.27	<.001
Preeclampsia	7 (14.58)	43 (43.87)	<.001	0.24	0.08-0.71	.01
HELLP[c]	1 (2.08)	4 (4.08)	.53			

[a]RM: remote monitoring.

[b]CC: conventional care.

[c]HELLP: hemolysis elevated liver enzymes and low platelets.

In order to investigate the influence of the maternal demographics and characteristics on the prenatal follow-up, a multiple linear regression analysis and a multivariate logistic regression analysis is performed. A detailed overview of these data is proved in Multimedia Appendix 1.

Delivery Outcomes: Comparison Between RM and CC

Delivery outcomes are shown in Table 3. For both uni- and multivariate analyses, in the RM group, the number of spontaneous start of the birth process was higher compared with CC group: 60.24% (29/48) versus 31.63% (31/98) and beta=3.25. Also, the number of inductions was lower in RM group compared with CC group: 25.00% (12/48) versus 48.98% (48/98) and beta=.36. Neonates in RM group did have a shorter length compared with the CC group when performed a multivariate analysis (beta=.23). Finally, neonates in the RM group, compared with CC group, were less likely to be admitted to the NIC department when performed a univariate analyses (10.42%, 5/48 vs 27.55%, 27/98) but not in multivariate analyses (beta=.34). Despite the significant differences in the start of the birth process, there are no differences in the mode of delivery between the two groups.

XSL•FO
RenderX

Table 3. Delivery outcomes.

Variable	Univariate analysis			Multivariate analysis		
	RM[a] group (n=48)	CC[b] group (n=98)	*P* value	RM versus no RM (beta)	95% CI for beta	*P* value
GA[c] delivery in weeks, mean (SD)	37.49 (2.52)	37.20 (3.20)	.94	−.21	−1.29 to 0.06	.85
**Start birth process, n (%)**						
Spontaneous	29 (60.42)	31 (31.63)	.001	3.25	1.36 to 7.78	.001
Induction	12 (25.00)	48 (48.98)	.006	.36	0.14 to 0.89	.03
Primary cesarean section	7 (14.54)	19 (19.39)	.48	.67	0.21 to 2.18	.51
**Mode of delivery, n (%)**						
Vaginal	32 (66.67)	58 (59.18)	.38	1.06	0.44 to 2.54	.90
Instrumental	4 (8.33)	8 (8.16)	.97	2.34	0.47 to 11.64	.30
Primary cesarean section	7 (14.54)	19 (19.39)	.48	.67	0.21 to 2.18	.51
Secondary cesarean section	5 (10.42)	13 (13.27)	.63	.49	0.11 to 2.10	.33
Birth weight in g, mean (SD)	3058.54 (692.60)	2953.09 (874.80)	.36	.11	−162.71 to 535.33	.29
Length in cm	49.53 (2.85)	48.33 (3.52)	.07	.23	0.02 to 3.45	.05
Apgar 1′, mean (SD)	8.11 (1.20)	7.91 (1.63)	.86	.08	−0.38 to 0.88	.43
Apgar 5′, mean (SD)	9.13 (0.80)	9.03 (1.27)	>.99	.06	−0.37 to 0.65	.59
Admission NIC[d], n (%)	5 (10.42)	27 (27.55)	.02	.34	0.10 to 1.14	.08

[a]RM: remote monitoring.

[b]CC: conventional care.

[c]GA: gestational age.

[d]NIC: neonatal intensive care.

In order to investigate the influence of the maternal demographics and characteristics on the delivery outcomes, a multiple linear regression analysis and multivariate logistic regression analysis is performed. A detailed overview of these data is proved in Multimedia Appendix 2.

Discussion

Principal Findings

We sought to determine whether RM was an added value to facilitate the prenatal follow-up and to improve the delivery outcomes in patients diagnosed with GHD. To our knowledge, this is the first publication about a prenatal follow-up program for pregnant women with GHD.

The findings show us a reduced appearance of preeclampsia, but an increased appearance of gestational hypertension in the group of women who received a prenatal RM program when compared with women who received CC. Women in the RM group, when compared with CC group, had a lower number of prenatal hospitalizations, prenatal hospitalizations until delivery, and their neonates were less likely to be admitted to the NIC department in univariate but not in multivariate analysis. In both analysis, spontaneous deliveries were more likely and inductions less likely to occur in the RM group when compared with CC group.

Strengths and Limitations

Despite the potential benefits, the use of RM in obstetrical care is still very limited and is not integrated into healthcare systems. The Commission of the European Communities has, in 2012, written an eHealth Action Plan [17] in which they foster a spirit of innovation in eHealth in Europe as the way forward to ensure better health. Our study is one of the first studies in the obstetrical care for women at risk for GHD which meets this requirement. Additionally, one of the strengths of this study is the fact that all patients had antenatal care and delivery in the same hospital with electronic medical records in line with administration files. Also, all patients had antenatal care according to uniform local management protocols. Finally, the percentage of missing data for RM group and CC group is 3.01% and 9.04% respectively, which is a low value.

Our study has three main limitations. First, the data collection was done retrospectively so selection bias cannot be excluded. Second, in CC group, there were more primigravida and women

XSL•FO
RenderX

who smoked during their pregnancy when compared with RM group. Although, our multivariate analysis did not show any influence of these parameters on our principal findings, nulliparous women are known to have a higher risk for the development of preeclampsia superimposed on chronic hypertension [1,13] and smoking during pregnancy carries adverse outcomes; however, a reduced risk of developing GHD in women who smoke is shown by many studies [1,3]. The last limitation is the interference from family doctors or community midwives which cannot be excluded.

Comparisons With Previous Trials

To our knowledge, this is the first publication about a prenatal follow-up program for pregnant women with GHD to date. There are a few publications about a RM program during prenatal follow-up in the management for pregnant women at risk for preterm labor or with the diagnosis of gestational diabetes mellitus. When looking at their maternal outcomes, the results of these studies are not in line with our findings. Compared with the usual care, these studies report no significant difference in prenatal hospitalizations [14] and mode of delivery [10,11] in RM group. When looking at the neonatal outcomes, some contradictions were found: the study of Corwin et al [9] and Morrison et al [12] states that infants born to monitored women were less likely to be admitted to a NIC compared with women without a RM follow-up program, which are in line with our findings. The Collaborative Home Uterine Monitoring Study Group [15] and Homko et al [16] did not find any difference between the two groups in neonatal hospitalization to the NIC. A side note which has to make is that some of the mentioned studies are dating back to 1995, which is in contradiction with the rapid technological advancements that have been made in the last decade.

Possible Explanations

A possible hypothesis of the differences in admission to the prenatal observational ward, admission to the NIC and the gestational outcomes is the hypothesis that preeclampsia is possibly a result of gestational hypertension or essential hypertension [18-20]. This may be due to the possibility to start or adjust an antihypertensive drugs therapy to reduce a high systolic or diastolic blood pressure which can be picked up by RM. There are some studies which mentioned a reduced risk of developing severe hypertension and preeclampsia associated with the use of antihypertensive drugs [21-24]. However, these results are in contradiction with the review of Duley [25], who states that antihypertensive drugs may be effective at reducing the risk of severe hypertension, but not of preeclampsia. Further examination of the influence of antihypertension drugs therapy on the development of severe hypertension or preeclampsia when moderate hypertension is diagnosed, is necessary to obtain clarification herein.

When women are diagnosed with preeclampsia, an induction of labor is often necessary for the prevent of further complications [26,27]. The explanation of more inductions in CC could be the higher number of women diagnosed with preeclampsia in this group. Gestational hypertension is not often a requirement to induce women, and a spontaneous onset of their labor is preferred. This can be the cause of the higher number of spontaneous start of labor in RM.

Additionally, our study shows that there are no differences in prenatal consults between RM and CC. These findings are in contradiction with the statement that medicalization of childbirth has gone too far, which arises from different angles [28-33]. Our study showed that adding RM devices to standard prenatal care does not mean an increase of total amount of echo's, CTG's or other prenatal consultations. In addition, RM opens the perspective to timely initiative and monitor antihypertensive treatments for gestational hypertension. As stated in the review of Gyselaers et al [34], offering RM to a high risk group allows timely identification of most cases of alarm events without increasing ambulatory or in-hospital interventions. This also opens perspectives to reverse the current evolution of antenatal interventions leading to more interventions and as such to ever increasing medicalized antenatal care.

Recommendations for Further Research

Although women in the RM group were invited for an extra prenatal consult to evaluate fetal and maternal wellbeing when events occurred, no statistical significant difference is present in prenatal consultations (total number of consultations, total number of CTG's, and total number of echo's) in the RM group versus the CC group. This indicates that RM does not cause extra prenatal consultations but, when further implemented, can ensure a reduction in this number when obstetricians and gynecologists are more familiar with this system. A study to evaluate the cost-effectiveness of a RM follow-up program needs to be performed later. Additionally, early detection of GHD in the monitoring group demonstrated the value of objective measurements of increase in blood pressure by a remote blood pressure monitoring device. The patients not receiving these devices relied on standard prenatal care, where a GHD mostly will be discovered by chance or when the patient comes to the hospital with self-reported complaints, for example, headache or blurred vision. In these cases, the degree of the GHD is often severe and an active management is necessary [1]. Recent resources showed that providing information about GHD enables women to spot signs and symptoms of these diseases. This leads to earlier diagnoses and management, and reduces morbidity and mortality rates [35]. It is possible that combining patient education and a remote prenatal follow-up program could make morbidity and mortality rates further decrease, but this requires further research. Finally, more research should be done to the influence of antihypertension drugs therapy on the development of severe hypertension or preeclampsia when moderate hypertension is diagnosed. When the effect of the medication is clarified, the added value of RM in the prenatal care of women diagnosed with GHD will be more apparent.

Conclusions

Prenatal RM follow-up is linked with an increased prevalence of a spontaneous start of the birth process, when compared with CC. This may relate to a trend for less maternal and neonatal hospitalizations in RM group compared with the CC group. This study illustrates that RM opens perspectives to timely initiate and monitor antihypertensive treatments for gestational

XSL•FO
RenderX

hypertension, and early identifications of alarm events without increasing ambulatory or in-hospital interventions. To our knowledge, this is the first publication about a prenatal follow-up program for pregnant women with GHD to date. Further examinations about the effect of a prenatal RM follow-up program for women at risk for the development of GHD needs to be done in a randomized controlled trial to confirm these results.

Acknowledgments

This study is part of the Limburg Clinical Research Program (LCRP) UHasselt-ZOL-Jessa, supported by the foundation Limburg SterkMerk (LSM), Hasselt University, Ziekenhuis Oost-Limburg, and Jessa Hospital. We like to thank NeleGeusens, Wilco Waaijer, and Thomas Reyskens for their participation in this project. No specific funding was provided for publication of this pilot study.

Conflicts of Interest

None declared.

Multimedia Appendix 1

Multivariable model for the prediction of prenatal follow-up using maternal demographics and characteristics.

[PDF File (Adobe PDF File), 36KB - mhealth_v5i3e25_app1.pdf]

Multimedia Appendix 2

Untitled.Supplementary file 2: Multivariable model for the prediction of gestational outcomes using maternal demographics and characteristics.

[PDF File (Adobe PDF File), 40KB - mhealth_v5i3e25_app2.pdf]

References

1. Singh R. Hypertensive Disorders in Pregnancy. Clinical Queries: Nephrology 2013;2:47-55.
2. Magee LA, Pels A, Helewa M, Rey E, von DP, Canadian Hypertensive Disorders of Pregnancy (HDP) Working Group. Diagnosis, evaluation, and management of the hypertensive disorders of pregnancy. Pregnancy Hypertens 2014 Apr;4(2):105-145 [FREE Full text] [doi: 10.1016/j.preghy.2014.01.003] [Medline: 26104418]
3. Gudnadóttir TA, Bateman BT, Hernádez-Díaz S, Luque-Fernandez MA, Valdimarsdottir U, Zoega H. Body Mass Index, Smoking and Hypertensive Disorders during Pregnancy: A Population Based Case-Control Study. PLoS One 2016;11(3):e0152187 [FREE Full text] [doi: 10.1371/journal.pone.0152187] [Medline: 27010734]
4. van Baaren GJ, Broekhuijsen K, van Pampus MG, Ganzevoort W, Sikkema JM, Woiski MD, HYPITAT-II Study Group. An economic analysis of immediate delivery and expectant monitoring in women with hypertensive disorders of pregnancy, between 34 and 37 weeks of gestation (HYPITAT-II). BJOG 2017;124(3):453-461. [doi: 10.1111/1471-0528.13957] [Medline: 26969198]
5. Cruz J, Brooks D, Marques A. Home telemonitoring in COPD: a systematic review of methodologies and patients' adherence. Int J Med Inform 2014 Apr;83(4):249-263. [doi: 10.1016/j.ijmedinf.2014.01.008] [Medline: 24529402]
6. Imelda N, Wendal P, Magann E. Telemedicine in Obstetrics. Clin Obstet gynecol 2013;56(3):422-433. [doi: 10.1097/GRF.0b013e318290fef0]
7. Giamouzis G, Mastrogiannis D, Koutrakis K, Karayannis G, Parisis C, Rountas C, et al. Telemonitoring in chronic heart failure: a systematic review. Cardiol Res Pract 2012;2012:410820 [FREE Full text] [doi: 10.1155/2012/410820] [Medline: 22720184]
8. Bolton CE, Waters CS, Peirce S, Elwyn G, EPSRCMRC Grand Challenge Team. Insufficient evidence of benefit: a systematic review of home telemonitoring for COPD. J Eval Clin Pract 2011 Dec;17(6):1216-1222. [doi: 10.1111/j.1365-2753.2010.01536.x] [Medline: 20846317]
9. Corwin MJ, Mou SM, Sunderji SG, Gall S, How H, Patel V, et al. Multicenter randomized clinical trial of home uterine activity monitoring: pregnancy outcomes for all women randomized. Am J Obstet Gynecol 1996 Nov;175(5):1281-1285. [Medline: 8942501]
10. Pérez-Ferre N, Galindo M, Fernández MD, Velasco V, Runkle I, de la Cruz MJ, et al. The outcomes of gestational diabetes mellitus after a telecare approach are not inferior to traditional outpatient clinic visits. Int J Endocrinol 2010;2010:386941 [FREE Full text] [doi: 10.1155/2010/386941] [Medline: 20628517]
11. Pérez-Ferre N, Galindo M, Fernández MD, Velasco V, de la Cruz MJ, Martín P, et al. A Telemedicine system based on Internet and short message service as a new approach in the follow-up of patients with gestational diabetes. Diabetes Res Clin Pract 2010 Feb;87(2):e15-e17. [doi: 10.1016/j.diabres.2009.12.002] [Medline: 20044162]

XSL•FO
RenderX

12. Morrison J, Bergauer NK, Jacques D, Coleman SK, Stanziano GJ. Telemedicine: cost-effective management of high-risk pregnancy. Manag Care 2001 Nov;10(11):42-6, 48 [FREE Full text] [Medline: 11761593]
13. Uzan J, Carbonnel M, Piconne O, Asmar R, Ayoubi J. Pre-eclampsia: pathophysiology, diagnosis, and management. Vasc Health Risk Manag 2011;7:467-474 [FREE Full text] [doi: 10.2147/VHRM.S20181] [Medline: 21822394]
14. Brown HL, Britton KA, Brizendine EJ, Hiett AK, Ingram D, Turnquest MA, et al. A randomized comparison of home uterine activity monitoring in the outpatient management of women treated for preterm labor. Am J Obstet Gynecol 1999 Apr;180(4):798-805. [Medline: 10203647]
15. Group TCHUMSC. A multicenter randomized controlled trial of home uterine monitoring: active versus sham device. The Collaborative Home Uterine Monitoring Study (CHUMS) Group. Am J Obstet Gynecol 1995 Oct;173(4):1120-1127. [Medline: 7485304]
16. Homko CJ, Deeb LC, Rohrbacher K, Mulla W, Mastrogiannis D, Gaughan J, et al. Impact of a telemedicine system with automated reminders on outcomes in women with gestational diabetes mellitus. Diabetes Technol Ther 2012 Jul;14(7):624-629 [FREE Full text] [doi: 10.1089/dia.2012.0010] [Medline: 22512287]
17. European Commission. 2012. Communication from the Commission to the European Parliament, the Council, the European Economic and Social Committee and the Committee of the Regions: eHealth Action Plan 2012 - 2020 - Innovative Healthcare for the 21st Century URL: http://ec.europa.eu/health//sites/health/files/ehealth/docs/com_2012_736_en.pdf [accessed 2017-02-23] [WebCite Cache ID 6oURgT1Gm]
18. Bramham K, Parnell B, Nelson-Piercy C, Seed PT, Poston L, Chappell LC. Chronic hypertension and pregnancy outcomes: systematic review and meta-analysis. BMJ 2014;348:g2301 [FREE Full text] [Medline: 24735917]
19. Berhan Y. No Hypertensive Disorder of Pregnancy; No Preeclampsia-eclampsia; No Gestational Hypertension; No Hellp Syndrome. Vascular Disorder of Pregnancy Speaks for All. Ethiop J Health Sci 2016 Mar;26(2):177-186 [FREE Full text] [Medline: 27222631]
20. Kintiraki E, Papakatsika S, Kotronis G, Goulis DG, Kotsis V. Pregnancy-Induced hypertension. Hormones (Athens) 2015;14(2):211-223 [FREE Full text] [Medline: 26158653]
21. Abalos E, Duley L, Steyn DW. Antihypertensive drug therapy for mild to moderate hypertension during pregnancy. Cochrane Database Syst Rev 2014(2):CD002252. [doi: 10.1002/14651858.CD002252.pub3] [Medline: 24504933]
22. Costa ML. Preeclampsia: Reflections on How to Counsel About Preventing Recurrence. J Obstet Gynaecol Can 2015 Oct;37(10):887-893. [Medline: 26606702]
23. Klocek M, Czarnecka D. Hypertension during pregnancy--how to manage effectively? Przegl Lek 2015;72(4):200-204. [Medline: 26455020]
24. Jiang N, Liu Q, Liu L, Yang WW, Zeng Y. The effect of calcium channel blockers on prevention of preeclampsia in pregnant women with chronic hypertension. Clin Exp Obstet Gynecol 2015;42(1):79-81. [Medline: 25864288]
25. Duley L. Pre-eclampsia, eclampsia, and hypertension. BMJ Clin Evid 2011 Feb 14;2011 [FREE Full text] [Medline: 21718554]
26. Zakiyah N, Postma MJ, Baker PN, van Asselt AD, IMPROvED Consortium. Pre-eclampsia Diagnosis and Treatment Options: A Review of Published Economic Assessments. Pharmacoeconomics 2015 Oct;33(10):1069-1082 [FREE Full text] [doi: 10.1007/s40273-015-0291-x] [Medline: 26048352]
27. George EM. New approaches for managing preeclampsia: clues from clinical and basic research. Clin Ther 2014 Dec 1;36(12):1873-1881 [FREE Full text] [doi: 10.1016/j.clinthera.2014.09.023] [Medline: 25450475]
28. Johanson R, Newburn M, Macfarlane A. Has the medicalisation of childbirth gone too far? BMJ 2002 Apr 13;324(7342):892-895 [FREE Full text] [Medline: 11950741]
29. Christiaens W, Van De Velde S, Bracke P. Pregnant women's fear of childbirth in midwife- and obstetrician-led care in Belgium and the Netherlands: test of the medicalization hypothesis. Women Health 2011 May;51(3):220-239. [doi: 10.1080/03630242.2011.560999] [Medline: 21547859]
30. Sedigh MS, Mirzaei NK, Ghazi TM. Ambivalence towards childbirth in a medicalized context: a qualitative inquiry among Iranian mothers. Iran Red Crescent Med J 2015 Mar;17(3):e24262 [FREE Full text] [doi: 10.5812/ircmj.24262] [Medline: 26019905]
31. Kuo S. A gender perspective on medicalized childbirth. Hu Li Za Zhi 2015 Feb;62(1):10-15. [Medline: 25631179]
32. Parry DC. "We wanted a birth experience, not a medical experience": exploring Canadian women's use of midwifery. Health Care Women Int 2008 Sep;29(8):784-806. [doi: 10.1080/07399330802269451] [Medline: 18726792]
33. Shaw JC. The medicalization of birth and midwifery as resistance. Health Care Women Int 2013;34(6):522-536. [doi: 10.1080/07399332.2012.736569] [Medline: 23514572]
34. Gyselaers W, Storms V, Grieten L. New technologies to reduce medicalization of prenatal care: a contradiction with realistic perspectives. Expert Rev Med Devices 2016 Aug;13(8):697-699. [doi: 10.1080/17434440.2016.1205484] [Medline: 27336237]
35. Wallis AB, Tsigas EZ, Saftlas AF, Sibai BM. Prenatal education is an opportunity for improved outcomes in hypertensive disorders of pregnancy: results from an Internet-based survey. J Matern Fetal Neonatal Med 2013 Nov;26(16):1565-1567. [doi: 10.3109/14767058.2013.797403] [Medline: 23927368]

XSL•FO
RenderX

Edited by G Eysenbach; submitted 26.08.16; peer-reviewed by M López-Coronado, I de la Torre, G Berntsen; comments to author 06.10.16; revised version received 03.11.16; accepted 14.01.17; published 09.03.17

Please cite as:
Lanssens D, Vandenberk T, Smeets CJP, De Cannière H, Molenberghs G, Van Moerbeke A, van den Hoogen A, Robijns T, Vonck S, Staelens A, Storms V, Thijs IM, Grieten L, Gyselaers W
Remote Monitoring of Hypertension Diseases in Pregnancy: A Pilot Study
JMIR Mhealth Uhealth 2017;5(3):e25
URL: http://mhealth.jmir.org/2017/3/e25/
doi:10.2196/mhealth.6552
PMID:28279948

©Dorien Lanssens, Thijs Vandenberk, Christophe JP Smeets, Hélène De Cannière, Geert Molenberghs, Anne Van Moerbeke, Anne van den Hoogen, Tiziana Robijns, Sharona Vonck, Anneleen Staelens, Valerie Storms, Inge M Thijs, Lars Grieten, Wilfried Gyselaers. Originally published in JMIR Mhealth and Uhealth (http://mhealth.jmir.org), 09.03.2017. This is an open-access article distributed under the terms of the Creative Commons Attribution License (http://creativecommons.org/licenses/by/2.0/), which permits unrestricted use, distribution, and reproduction in any medium, provided the original work, first published in JMIR mhealth and uhealth, is properly cited. The complete bibliographic information, a link to the original publication on http://mhealth.jmir.org/, as well as this copyright and license information must be included.

XSL•FO
RenderX
